# Association Between Serum Micronutrients and Advanced CKM Syndrome: An Interpretable Machine Learning‐Based Study

**DOI:** 10.1002/fsn3.71059

**Published:** 2025-10-22

**Authors:** Yi Ou, Xinyi Shao, Yidian Fu, Xiang Qu, Yuan Zhan, Qian Liu, Jin Chen, Aijun Chen, Genlong Bai, Jingbo Zhang

**Affiliations:** ^1^ Department of Dermatology The First Affiliated Hospital of Chongqing Medical University Chongqing China; ^2^ Graduate School of Hebei Medical University Shijiazhuang Hebei China; ^3^ Department of Cardiovascular Surgery Nanfang Hospital, Southern Medical University Guangzhou China; ^4^ Department of Respiratory and Critical Care Medicine The First Affiliated Hospital of Chongqing Medical University Chongqing China

**Keywords:** advanced CKM syndrome, machine learning, micronutrients, NHANES, SHAP

## Abstract

CKM is a common systemic disorder characterized by pathological and physiological interactions between the cardiovascular system. In recent years, the intake of micronutrients has been considered to be associated with CVD, CKD, and other conditions. However, the association between serum micronutrients and advanced CKM syndrome is still unclear. This study utilized data from the NHANES to analyze 6 serum micronutrients and employed the Boruta algorithm for feature selection. Subsequently, the risk of advanced CKM syndrome was predicted using 7 ML models. Additionally, SHAP and PDPs were employed to analyze the effects of serum micronutrients on advanced CKM syndrome. Results indicate that lower levels of β‐carotene, α‐tocopherol, lycopene, vitamin C, and 25(OH)D are associated with an increased risk of advanced CKM syndrome (all *p* < 0.05). The LGBM model exhibited the best performance in predicting the risk of advanced CKM syndrome, while SHAP and PDPs analyses show that the low level of 25(OH)D is the main potential risk factor. The synergistic effects indicated that managing serum 25(OH)D, vitamin C, and lycopene levels may play a vital role in the control of advanced CKM syndrome. This study systematically evaluated the relationship between serum micronutrients and the risk of advanced CKM syndrome using multiple models and ML methods for the first time. It identified the decrease of 25(OH)D, lycopene, and vitamin C as the main potential risk factors. These findings provide new evidence for the etiology of advanced CKM syndrome and public health interventions.

## Graphical abstract

This study explored the association between serum micronutrients and advanced Cardiovascular–Kidney–Metabolic (CKM) syndrome. This condition involves combined damage to the heart, kidneys, and metabolism.

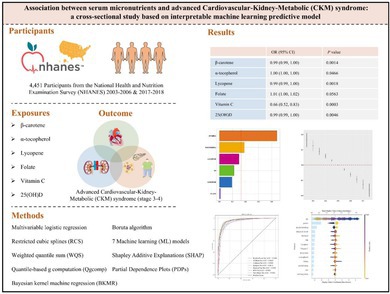

**Abbreviations**
AHA: American Heart Association
AUC: area under the curve
BKMR: Bayesian kernel machine regression
BMI: body mass index
CB: CatBoost
CIs: confidence intervals
CKD: chronic kidney disease
CKM: cardiovascular–kidney–metabolic syndrome
CVD: cardiovascular disease
DT: Decision Tree
HPLC: high‐performance liquid chromatography
IQR: interquartile range
KNN: k‐nearest neighbors
LC–MS/MS: liquid chromatography coupled with tandem mass spectrometry
LGBM: Light Gradient Boosting Machine
MCMC: Markov Chain Monte Carlo
MetS: metabolic syndrome
ML: machine learning
NB: naïve Bayes classifier
NCHS: National Center for Health Statistics
NHANES: National Health and Nutrition Examination Survey
ORs: odds ratios
PDPs: partial dependence plots
PIP: posterior inclusion probability
PIR: poverty income ratio
PUFA: polyunsaturated fatty acid
Qgcomp: quantile‐based g computation
RCS: restricted cubic splines
RF: random forest classifier
RNS: reactive nitrogen species
SD: standard deviation
SHAP: Shapley additive explanations
SMOTE: Synthetic Minority Oversampling Technique
VIF: variance inflation factor
WQS: weighted quantile sum
XGB: eXtreme Gradient Boosting

## 1 Introduction

CKM syndrome, a newly defined systemic condition, is attributable to the complex interactions among various metabolic disorders, chronic cardiovascular disease (CVD), and chronic kidney disease (CKD) (Aggarwal et al. 2024; Ndumele, Neeland, et al. 2023; Ndumele, Rangaswami, et al. 2023). To enhance the prevention and management strategies for CKM syndrome, the disease is stratified into stages ranging from Stage 0 (no risk factors) to Stage 4 (established CVD) according to risk factors and established disease. Recent research indicates that CKM syndrome is highly prevalent in the population, with nearly 90% of individuals meeting the criteria for CKM syndrome Stage 1 or higher during the period from 2011 to 2020. Notably, 14.6% of American adults are categorized as having advanced CKM syndrome (Stage 3 or 4) (Aggarwal et al. 2024).

Current research suggests that drinking, smoking, exercise, bad eating habits, and other living habits affect the risk of obesity, diabetes, CKD, and CVD (Chudasama and Khunti 2023; Kim et al. 2024; Zhang et al. 2024, 2021). Among these risk factors, poor diet consistently ranked as the second leading risk factor for CVD, diabetes, and the third or fourth for CKD (Arnett et al. 2019; Group 2024). Therefore, implementing dietary interventions may help to mitigate the risk of CKM syndrome development. Micronutrients are crucial for regulating oxidative stress and inflammation, both of which are CVD, CKD, diabetes, and other major driving factors (Shenkin 2006). Research has demonstrated that both fat‐soluble vitamins A and E (α‐tocopherol) and water‐soluble vitamin C have anti‐inflammatory properties. These vitamins interact with reactive nitrogen species (RNS) and several free radicals to decrease polyunsaturated fatty acid (PUFA) peroxidation, thus protecting cell membrane phospholipids and plasma lipoproteins and benefiting glutathione status and antioxidant capacity (Gombart et al. 2020). Previous studies have likewise shown decreased serum micronutrient levels in patients with CVD, diabetes, CKD, and metabolic syndrome (MetS) (Abboud et al. 2023; Aune et al. 2018; Pittas et al. 2023; Wu et al. 2023). However, there is currently no study examining the association between advanced CKM syndrome, a comprehensive disease state, and serum micronutrients.

In recent years, the emergence of machine learning (ML) has provided opportunities for better recognition of complex clinical patterns for disease risk prediction and mechanism exploration. Unlike traditional regression models, ML algorithms allow processing high‐dimensional data and unveiling non‐linear relationships, which may be crucial for the prediction of CKM syndrome, a multifactorial disease (Christodoulou et al. 2019; Lundberg et al. 2020). Building on this, we integrated data from the National Health and Nutrition Examination Survey (NHANES) ranging from 2003 to 2006 and 2017 to 2018 and conducted several traditional analytical methods, including multivariable logistic regression, restricted cubic splines (RCS), weighted quantile sum (WQS) regression, quantile‐based *g* computation (Qgcomp) regression, and Bayesian kernel machine regression (BKMR) regression models to investigate the relationship of the serum micronutrients with advanced CKM syndrome. A multimodal ML model combining demographic variables, clinical indicators, and six serum micronutrients has been developed. We used the Boruta algorithm for feature selection while optimizing prediction performance through the Light Gradient Boosting Machine (LGBM) model. In addition, Shapley Additive Explanations (SHAP) and Partial Dependence Plots (PDPs) are used to elucidate the independence and interaction of key risk factors. This study not only provides ML‐based epidemiological evidence for the association between serum micronutrients and advanced CKM syndrome, but also lays a methodological foundation for precise prevention and public health intervention strategies.

## 2 Methods

### 2.1 Selection of Study Population

The NHANES represents a comprehensive investigation biennially to assess the health/nutritional conditions of the US non‐institutionalized demographic civilians. Each iteration of the NHANES is sanctioned by the Ethics Review Board of the National Center for Health Statistics (NCHS), and all participants are required to provide written informed consent. Initially, 29,724 participants were enrolled (NHANES survey 2003–2006 and 2017–2018). To maintain precision and dependability, particular exclusion criteria for participants' selection were used, including (1) age below 20 (*n* = 14,135); (2) missing CKM‐related data (*n* = 9509); (3) those who did not have complete serum micronutrients data (*n* = 1007); and (4) missing measurements of one or more covariates (*n* = 622). Finally, the sample size included in the study was 4451 participants after screening (Figure S1).

### 2.2 Assessments of CKM Syndrome

CKM syndrome is defined by the simultaneous presence of subclinical or clinical CVD, CKD, and metabolic disorders (Table S1). In order to reflect the varying degrees of clinical severity associated with CKM syndrome, the participants were categorized into four separate stages. Stage 0 indicates the absence of CKM risk factors, while Stage 1 is defined by the presence of excess or dysfunctional adiposity alone. Stage 2 encompasses supplementary metabolic risk factors alongside CKD. Stage 3 is characterized by the presence of subclinical CVD, while Stage 4 is distinguished by the emergence of clinical manifestations of CVD in CKM (Table S2). Stages 3 and 4 were combined and classified as advanced CKM syndrome. The 10‐year CVD risk for individuals was evaluated utilizing the American Heart Association (AHA) PREVENT equations (Table S3) (Zhu et al. 2024).

### 2.3 Assessment of Micronutrients in Serum

Details of the collection and processing procedures are documented in the NHANES Laboratory Procedure Manual (Zipf et al. 2013). To assess the serum concentrations of specific micronutrients, a range of analytical methodologies was implemented. Isocratic liquid chromatography coupled with tandem mass spectrometry (LC–MS/MS) was employed for the quantification of 25(OH)D. High‐performance liquid chromatography (HPLC) equipped with electrochemical detection at 650 mV was utilized to measure vitamin C levels. Additionally, HPLC with photodiode array detection was applied for the assessment of α‐tocopherol, β‐carotene, and lycopene. The Roche Cobas 6000 analyzer, in conjunction with the FerroZine reagent, was utilized for the assessment of iron levels. During the 2003–2006 cycles, serum folate was quantified using the Bio‐Rad Quantaphase II Folate radioassay, whereas the 2017–2018 cycles used isotope‐dilution LC–MS/MS. The two methods' serum folate level measurements have been corrected for calibration bias within the dataset (Liu et al. 2022).

### 2.4 Covariables Definition

In the present investigation, various covariates were gathered from the NHANES datasets spanning 2003–2006 and 2017–2018. These covariates encompassed gender, age, race/ethnicity, educational attainment, family poverty income ratio (PIR), marital status, smoking habits, and alcohol consumption status.

Specifically, gender was divided into male and female categories. Race/ethnicity was grouped as Mexican American, non‐Hispanic Black/White, and other ethnicities. Marital status was classified into married, never married, living with a partner, and other statuses. The assessment of alcohol consumption was based on the survey question: “In any 1 year, (Have you/Has SP) had at least 12 drinks of any type of alcoholic beverage?” Respondents who answered “Yes” were identified as current alcohol users. Smoking status was defined as “yes” for participants who had smoked at least 100 cigarettes in their lifetime.

### 2.5 Traditional Regression Models

#### 2.5.1 Construction of Multivariable Regression Model and RCS Model

In order to evaluate the association between continuous serum micronutrient levels and advanced CKM syndrome, we utilized multivariable regression techniques alongside RCS models to derive odds ratios (ORs) and 95% confidence intervals (CIs). Our analysis comprised three distinct models: Model 1, which was unadjusted; Model 2, which incorporated essential demographic factors such as age, gender, and race/ethnicity; and Model 3, the fully adjusted model that accounted for all covariates. In the RCS analysis, we set up three knots at the 10th, 50th, and 90th percentiles of the serum micronutrient levels to determine whether there is a nonlinear association between serum micronutrient levels and advanced CKM syndrome.

#### 2.5.2 WQS & Qgcomp & BKMR Regression Analyses

We used the WQS regression analysis to evaluate the combined impact of serum micronutrients on advanced CKM syndrome and identified the corresponding weights (Model 3; training and validation sets: 40% and 60%). We also computed the WQS index using serum micronutrient weights in the training subset (10,000 bootstrap). The validation subset was used to assess the relationship between the WQS index and the risk of advanced CKM syndrome.

To address the limitation of the WQS regression model, which can only measure exposures related to outcomes in the same direction, we explored the Qgcomp regression model. This model allows for the calculation of positive and negative weights of each serum micronutrient without assuming uniformity of direction. We applied the Qgcomp regression model to estimate the weights of each serum micronutrient (without bootstrap) and the ORs of the joint effect (with 10,000 bootstrap iterations).

Ultimately, BKMR models were employed to examine the collective impact of six serum micronutrients on advanced CKM syndrome while considering possible nonlinear relationships and interaction effects. All serum micronutrient values were subjected to logarithmic transformation. The BKMR models underwent fitting with 25,000 iterations utilizing the Markov Chain Monte Carlo (MCMC) algorithm, with adjustments made for all relevant covariates. The effect of each serum micronutrient on advanced CKM syndrome was assessed by computing the posterior inclusion probability (PIP), where a threshold of 0.5 was established to signify statistical significance.

### 2.6 Machine Learning Models

#### 2.6.1 Selection and Assessment of Features

In the present study, the dataset initially comprised 17 variables, which were used as features in the following ML models. The complex nature of high‐dimensional data may adversely impact ML algorithm performance. To address this, prior to model development, the Boruta algorithm—a wrapper‐based feature selection method—was used to identify the most robust/critical factors to be included in the model. Next, Pearson's correlation analysis was conducted to assess the correlations of the selected features. We used variance inflation factor (VIF) to evaluate multicollinearity between selected features (Table S4). Features with minimal contribution or significant multicollinearity are excluded from the model.

#### 2.6.2 Construction and Evaluation of the ML Models

The Synthetic Minority Oversampling Technique (SMOTE) preprocessing algorithm was used in this study to reduce the imbalance of samples between the participants with advanced CKM syndrome and those without advanced CKM syndrome (Figure S2). Subsequently, the dataset after SMOTE was randomly divided into two subsets, comprising 80% designated as the training set and 20% allocated as the testing set. Next, 7 ML models were used to model construction on the basis of the training set: eXtreme Gradient Boosting (XGBoost, XGB), random forest classifier (RF), CatBoost (CB), LGBM, Decision Tree (DT), k‐nearest neighbors (KNN), and naïve Bayes classifier (NB). All 7 ML models' hyper‐parameters were optimized using grid search and 5‐fold cross‐validation in the training datasets. The detailed hyperparameter configurations of all models are listed in Table S5. The calculation formulas for the ML model metrics are listed in Table S6.

The AUC values, specificity, and sensitivity were utilized to compare 7 ML models' performance and to select the most appropriate model for further interpretability. Moreover, 1000 Bootstrap resampling was conducted to estimate the distribution of AUCs of all 7 models. Each confusion matrix of ML models and the calculation methods of several confusion matrix metrics are shown in Table S7.

#### 2.6.3 Two Interpretable Methods for ML Models

After the best performance ML model was selected, we conducted 2 interpretative methods, including the SHAP method and the PDPs method, to further investigate the effect of features on advanced CKM syndrome risk. SHAP is a novel model explanation method addressing the black‐box issues of ML models. To determine the significance of various features, both the SHAP summary plot and the SHAP bar plot were utilized. The implementation of SHAP in personalized decision‐making illustrates the potential of ML models in facilitating the creation of tailored care strategies (Lundberg et al. 2020). PDPs were utilized to enhance the understanding of the connections between the desired outcome and the chosen input variables through the application of nonlinear regression techniques. Here, we conducted both one‐dimensional and two‐dimensional analyses to illustrate the single effect and binary effect of selected features on the predicted outcome (Zhang et al. 2025).

### 2.7 Statistical Analysis

In the present study, participants were categorized into four distinct groups based on the quartiles of their serum micronutrient concentrations (Q1–Q4). For continuous variables with a normal distribution, the mean ± standard deviation (SD) was reported. Variables exhibiting a non‐normal distribution were summarized using the median along with the interquartile range (IQR). Categorical data was represented through frequency counts and corresponding percentages. According to NHANES analytic guidelines, complex sampling design and sampling weights were considered in baseline, multivariable logistic regression, and RCS analyses (Detail: https://wwwn.cdc.gov/nchs/nhanes/analyticguidelines.aspx). However, we didn't use the weighting and complex survey design in WQS, Qgcomp, BKMR, and ML‐related analyses due to the nature of these methods. All statistical analyses were performed using R software (version 4.3.1) and EmpowerStats (version 4.1), with two‐sided tests applied. A *p*‐value < 0.05 was considered to indicate statistical significance.

## 3 Results

### 3.1 Demographic Characteristics

Taking into consideration the complex survey design of NHANES, all results were weighted to provide a national estimate of noninstitutionalized US civilian residents. Table 1 displays the characteristics of 4451 adults from NHANES 2003 to 2006 and 2017 to 2018. The sample includes an average age of 46.0 years, with 47.6% male and 52.4% female. The majority (71.9%) were non‐Hispanic White, and 57.8% had at least a college education. A significant proportion (50.0%) were nonsmokers, and 70.7% reported alcohol consumption. Significant differences were observed between the advanced CKM syndrome group and those in the low CKM stage regarding age, gender, race, education level, marital status, smoking, and alcohol status. Serum levels of all micronutrients, except for vitamin C and 25‐hydroxyvitamin D (25(OH)D), showed significant differences between the two groups (*p* < 0.001).

**TABLE 1:** Baseline characteristics of study participants in the NHANES datasets 2003–2006 and 2017–2018.

Characteristic	Participants^a^			*p*
	Total (*N* = 4451)	With advanced CKM syndrome (*N* = 953)	Without advanced CKM syndrome (*N* = 3498)	
Age, mean ± SD	46.0 (45.1, 47.0)	68.9 (67.8, 70.0)	42.4 (41.6, 43.1)	< 0.001
PIR, mean ± SD	3.1 (3.0, 3.2)	2.7 (2.5,2.8)	3.2 (3.1, 3.2)	< 0.001
β‐carotene, mean ± SD	19.4 (18.1, 20.8)	20.8 (19.3, 22.3)	19.2 (17.7, 20.7)	0.098
α‐tocopherol, mean ± SD	1283.7 (1255.7, 1311.6)	1507.5 (1445.8, 1569.3)	1247.5 (1221.0, 1274.0)	< 0.001
Lycopene, mean ± SD	42.4 (41.5, 43.3)	34.2 (32.5, 35.8)	43.8 (42.7, 44.8)	< 0.001
Folate, mean ± SD	14.4 (13.9, 15.0)	19.5 (17.7, 21.2)	13.6 (13.1, 14.1)	< 0.001
Vitamin C, mean ± SD	0.9 (0.9, 0.9)	0.9 (0.9, 1.0)	0.9 (0.9, 0.9)	0.032
25(OH)D, mean ± SD	65.7 (63.4, 68.0)	64.3 (61.2, 67.3)	65.9 (63.5, 68.3)	0.233
Gender, *N* (%)				
Male	2134 (47.6) [45.9, 49.3]	575 (55.1) [51.4, 58.8]	1559 (46.4) [44.5, 48.3]	< 0.001
Female	2317 (52.4) [50.7, 54.1]	378 (44.9) [41.2, 48.6]	1939 (53.6) [51.7, 55.5]	
Race and ethnicity^b^, *N* (%)				
Hispanic	1008 (11.6) [9.4, 14.2]	152 (5.5) [3.6, 8.2]	856 (12.6) [10.3, 15.3]	< 0.001
Non‐Hispanic White	2228 (71.9) [68.1, 75.5]	573 (78.7) [73.4, 83.1]	1655 (70.8) [66.9, 74.5]	
Non‐Hispanic Black	913 (10.3) [8.5, 12.5]	189 (11.0) [8.3, 14.6]	724 (10.2) [8.4, 12.4]	
Other	302 (6.2) [5.1, 7.4]	39 (4.9) [3.0, 7.7]	263 (6.4) [5.2, 7.8]	
Educational, *N* (%)				
Below than high school	1124 (15.7) [14.0, 17.6]	317 (23.7) [20.3, 27.6]	807 (14.4) [12.7, 16.2]	< 0.001
High school/equivalent	1089 (26.5) [24.7, 28.4]	258 (30.6) [26.2, 35.5]	831 (25.9) [24.1, 27.7]	
Greater than high school	2238 (57.8) [55.0, 60.5]	378 (45.6) [40.0, 51.4]	1860 (59.7) [57.1, 62.3]	
Marital status^c^, *N* (%)				
Married	2436 (57.4) [55.0, 59.8]	531 (59.0) [54.6, 63.3]	1905 (57.2) [54.5, 59.8]	< 0.001
Never married	689 (15.3) [13.5, 17.4]	41 (3.4) [2.2, 5.1]	642 (17.2) [15.2, 19.5]	
Living with partner	389 (9.1) [7.5, 11.1]	22 (3.3) [2.0, 5.4]	367 (10.1) [8.2, 12.3]	
Others	943 (18.1) [16.5, 19.9]	359 (34.3) [30.0, 39.0]	584 (15.5) [13.9, 17.3]	
Smoking status, *N* (%)				
No	2262 (50.0) [47.4, 52.6]	367 (37.9) [34.0, 45.3]	1895 (52.0) [49.2, 54.8]	< 0.001
Yes	2189 (50.0) [47.4, 52.6]	586 (62.1) [58.0, 66.0]	1603 (48.0) [45.2, 50.8]	
Alcohol drinking, *N* (%)				
No	1529 (29.3) [26.4, 32.2]	386 (39.6) [34.1, 45.3]	1143 (27.6) [24.8, 30.6]	< 0.001
Yes	2922 (70.7) [67.8, 73.6]	567 (60.4) [54.7, 65.9]	2355 (72.4) [69.4, 75.2]	
Survey cycle, *N* (%)				
2003–2004	1706 (36.4) [32.4, 40.6]	395 (36.7) [29.5, 44.5]	1311 (36.3) [32.4, 40.5]	0.055
2005–2006	1794 (39.8) [36.0, 43.6]	383 (45.2) [37.0, 53.7]	1411 (38.9) [35.2, 42.7]	
2017–2018	951 (23.9) [21.3, 26.7]	175 (18.2) [13.8, 23.5]	776 (24.8) [22.0, 27.8]	

Abbreviations: CKM, Cardiovascular–Kidney–Metabolic; PIR, poverty impact ratio; SD, standard deviation.

^a^ Continuous data are presented as weighted mean (95% CI), while categorized data are shown as unweighted number (weighted percentage) [95% CI] unless otherwise specified.

^b^ Race and ethnicity were self‐reported.

^c^ Included widowed, divorced, or separated.

### 3.2 Associations of Serum Micronutrients With Advanced CKM Syndrome

In Table 2, we conducted multivariate logistic regression analysis using serum micronutrients as continuous variables and their quartiles as categorical variables. The consistent results showed that as serum levels of serum β‐carotene, α‐tocopherol, lycopene, vitamin C, and 25(OH)D increased, the overall risk of advanced CKM syndrome decreased (all *p* < 0.05). In Model 3, the results of multiple logistic regression showed that as the concentration of six serum micronutrients increased, the risk of advanced CKM syndrome decreased for both continuous and categorical variables.

**TABLE 2:** The association of serum micronutrients with advanced CKM syndrome among participants in the NHANES 2003–2006 and 2017–2018.

Serum micronutrients	Model 1^a^		Model 2^b^		Model 3^c^	
	OR (95% CI)	*p*	OR (95% CI)	*p*	OR (95% CI)	*p*
**β‐carotene (μg/dL)**						
Per 1 unit increase	**1.00 (1.00, 1.01)**	**0.0007**	**0.99 (0.99, 1.00)**	**< 0.0001**	**0.99 (0.99, 1.00)**	**0.0014**
Quartiles						
Q1 (0.49 to < 7.38)	1 (Ref.)		1 (Ref.)		1 (Ref.)	
Q2 (7.38 to < 12.69)	1.19 (0.96, 1.47)	0.1182	0.83 (0.61, 1.12)	0.2261	0.89 (0.65, 1.21)	0.4604
Q3 (12.70 to < 23.38)	**1.47 (1.19, 1.80)**	**0.0003**	**0.62 (0.46, 0.84)**	**0.0021**	**0.69 (0.50, 0.94)**	**0.0186**
Q4 (23.40 to < 343.04)	**1.54 (1.25, 1.89)**	**< 0.0001**	**0.45 (0.33, 0.61)**	**< 0.0001**	**0.58 (0.42, 0.82)**	**0.0017**
**α‐tocopherol (μg/dL)**						
Per 1 unit increase	**1.00 (1.00, 1.00)**	**< 0.0001**	**1.00 (1.00, 1.00)**	**0.0078**	**1.00 (1.00, 1.00)**	**0.0466**
Quartiles						
Q1 (41.0 to < 930.0)	1 (Ref.)		1 (Ref.)		1 (Ref.)	
Q2 (931.0 to < 1159.0)	1.20 (0.96, 1.51)	0.1151	**0.71 (0.54, 0.93)**	**0.0136**	0.72 (0.51, 1.01)	0.0585
Q3 (1160.0 to < 1497.0)	**1.59 (1.28, 1.97)**	**< 0.0001**	**0.62 (0.46, 0.82)**	**0.0009**	**0.66 (0.50, 0.88)**	**0.0053**
Q4 (1500.0 to < 6905.0)	**2.59 (2.10, 3.18)**	**< 0.0001**	**0.59 (0.44, 0.80)**	**0.0006**	**0.65 (0.48, 0.88)**	**0.0060**
**Lycopene (μg/dL)**						
Per 1 unit increase	**0.97 (0.97, 0.97)**	**< 0.0001**	**0.99 (0.98, 0.99)**	**0.0001**	**0.99 (0.99, 1.00)**	**0.0018**
Quartiles						
Q1 (0.7 to < 26.59)	1 (Ref.)		1 (Ref.)		1 (Ref.)	
Q2 (26.6 to < 37.83)	**0.45 (0.37, 0.54)**	< 0.0001	**0.71 (0.54, 0.93)**	**0.0136**	**0.74 (0.56, 0.98)**	**0.0352**
Q3 (37.9 to < 51.83)	**0.33 (0.27, 0.40)**	< 0.0001	**0.62 (0.46, 0.82)**	**0.0009**	**0.66 (0.50, 0.88)**	**0.0053**
Q4 (51.9 to < 151.0)	**0.25 (0.20, 0.31)**	< 0.0001	**0.59 (0.44, 0.80)**	**0.0006**	**0.65 (0.48, 0.88)**	**0.0060**
**Folate (ng/mL)**						
Per 1 unit increase	**1.05 (1.04, 1.06)**	**< 0.0001**	1.01 (1.00, 1.02)	0.0986	1.01 (1.00, 1.02)	0.0563
Quartiles						
Q1 (0.7 to < 8.5)	1 (Ref.)		1 (Ref.)		1 (Ref.)	
Q2 (8.52 to < 12.0)	**1.32 (1.06, 1.65)**	**0.0145**	0.86 (0.62, 1.18)	0.3484	0.89 (0.64, 1.23)	0.4748
Q3 (12.1 to < 17.0)	**1.38 (1.11, 1.73)**	**0.0039**	**0.63 (0.46, 0.87)**	**0.0051**	**0.69 (0.50, 0.95)**	**0.0232**
Q4 (17.5 to < 294.7)	**2.65 (2.16, 3.26)**	**< 0.0001**	**0.65 (0.48, 0.90)**	**0.0080**	**0.72 (0.52, 0.99)**	**0.0418**
**Vitamin C (μmol/L)**						
Per 1 unit increase	1.10 (0.93, 1.28)	0.2594	**0.59 (0.47, 0.74)**	**< 0.0001**	**0.66 (0.52, 0.83)**	**0.0003**
Quartiles						
Q1 (0.01 to < 0.6)	1 (Ref.)		1 (Ref.)		1 (Ref.)	
Q2 (0.6 to < 0.94)	0.83 (0.67, 1.01)	0.0688	**0.72 (0.54, 0.98)**	**0.0335**	0.81 (0.60, 1.10)	0.1811
Q3 (0.94 to < 1.19)	0.83 (0.68, 1.01)	0.0689	**0.65 (0.48, 0.88)**	**0.0044**	0.76 (0.56, 1.03)	0.0730
Q4 (1.2 to < 3.44)	1.06 (0.87, 1.29)	0.5712	**0.49 (0.36, 0.66)**	**< 0.0001**	**0.56 (0.41, 0.76)**	**0.0002**
**25(OH)D (ng/mL)**						
Per 1 unit increase	1.00 (1.00, 1.00)	0.5406	**0.99 (0.99, 1.00)**	**0.0008**	**0.99 (0.99, 1.00)**	**0.0046**
Quartiles						
Q1 (9.1 to < 43.3)	1 (Ref.)		1 (Ref.)		1 (Ref.)	
Q2 (43.4 to < 59.1)	1.00 (0.82, 1.23)	0.9861	**0.72 (0.54, 0.98)**	**0.0368**	0.76 (0.56, 1.03)	0.0819
Q3 (59.2 to < 75.1)	0.99 (0.80, 1.21)	0.9006	**0.59 (0.43, 0.81)**	**0.0013**	**0.63 (0.45, 0.87)**	**0.0049**
Q4 (75.3 to < 238.0)	1.03 (0.84, 1.26)	0.7901	**0.54 (0.40, 0.74)**	**0.0001**	**0.57 (0.42, 0.79)**	**0.0006**

*Note:* The bold value presented in Table 2 means statistically significance (*p*‐value < 0.05).

^a^ Model 1 was unadjusted.

^b^ Model 2 was adjusted for age, gender, and race/ethnicity.

^c^ Model 3 was further adjusted for educational level, marital status, PRI, smoking status, alcohol drinking status, and survey cycles.

**FIGURE 1:**
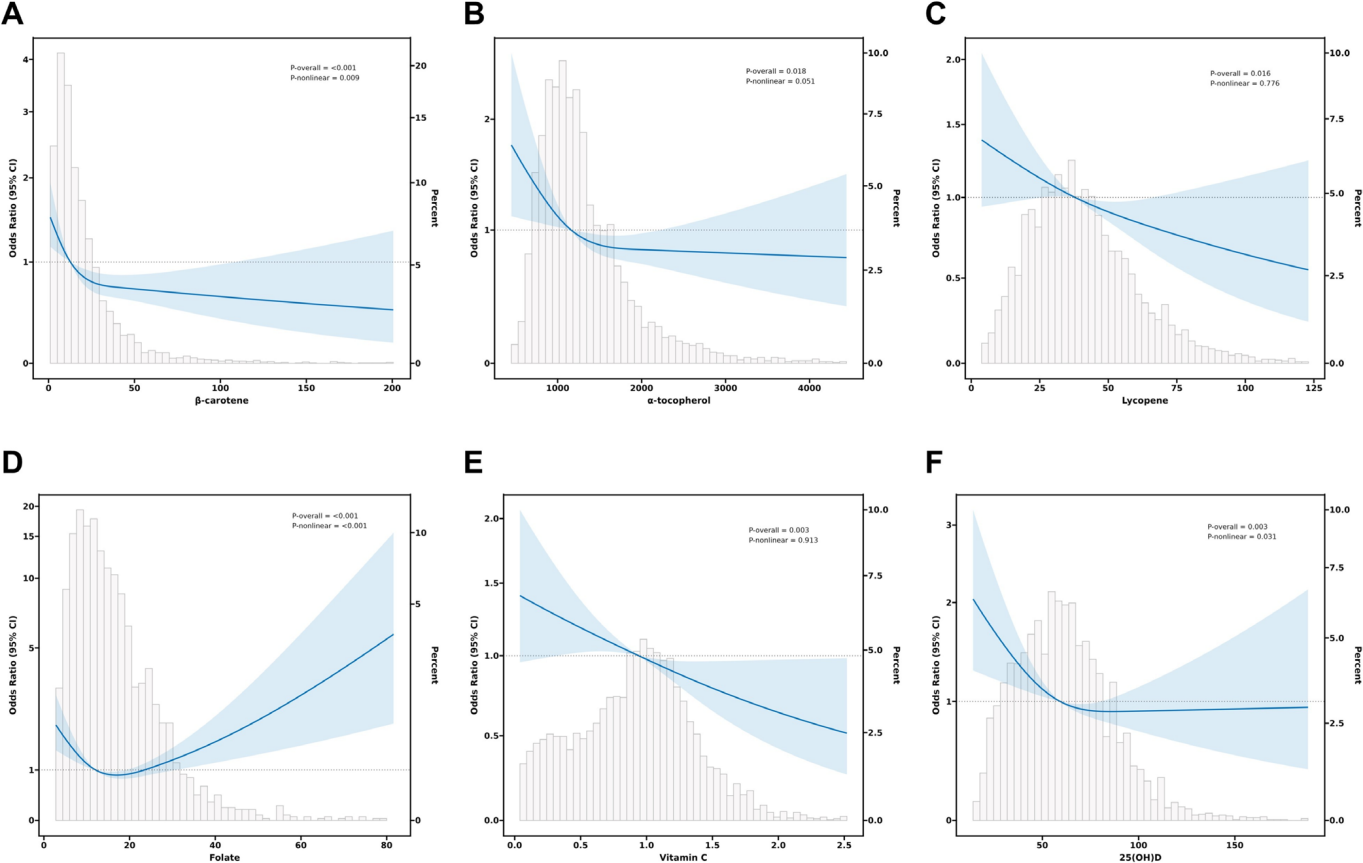
RCS analysis for association between serum micronutrients and advanced CKM syndrome. Multivariable adjusted restricted cubic splines curve for associations of advanced CKM syndrome participants and (A) β‐carotene, (B) α‐tocopherol, (C) lycopene, (D) folate, (E) vitamin C, and (F) 25(OH)D. The solid and shaded areas represent the estimated values and their corresponding 95% CI.

Additionally, our study explored the potential relationship between six serum micronutrients and the risk of advanced CKM syndrome using RCS curves. The results significantly demonstrated a non‐linear association between β‐carotene, folate, and 25(OH)D and advanced CKM syndrome (all *p*_nonlinear_ < 0.05). In contrast, the associations between serum lycopene, vitamin C, and α‐tocopherol with the risk of advanced CKM syndrome were linear (*p*_overall_ < 0.05, *p*_nonlinear_ > 0.05) (Figure 1).

### 3.3 WQS & Qgcomp Regression Analyses and BKMR Regression Analyses

**FIGURE 2:**
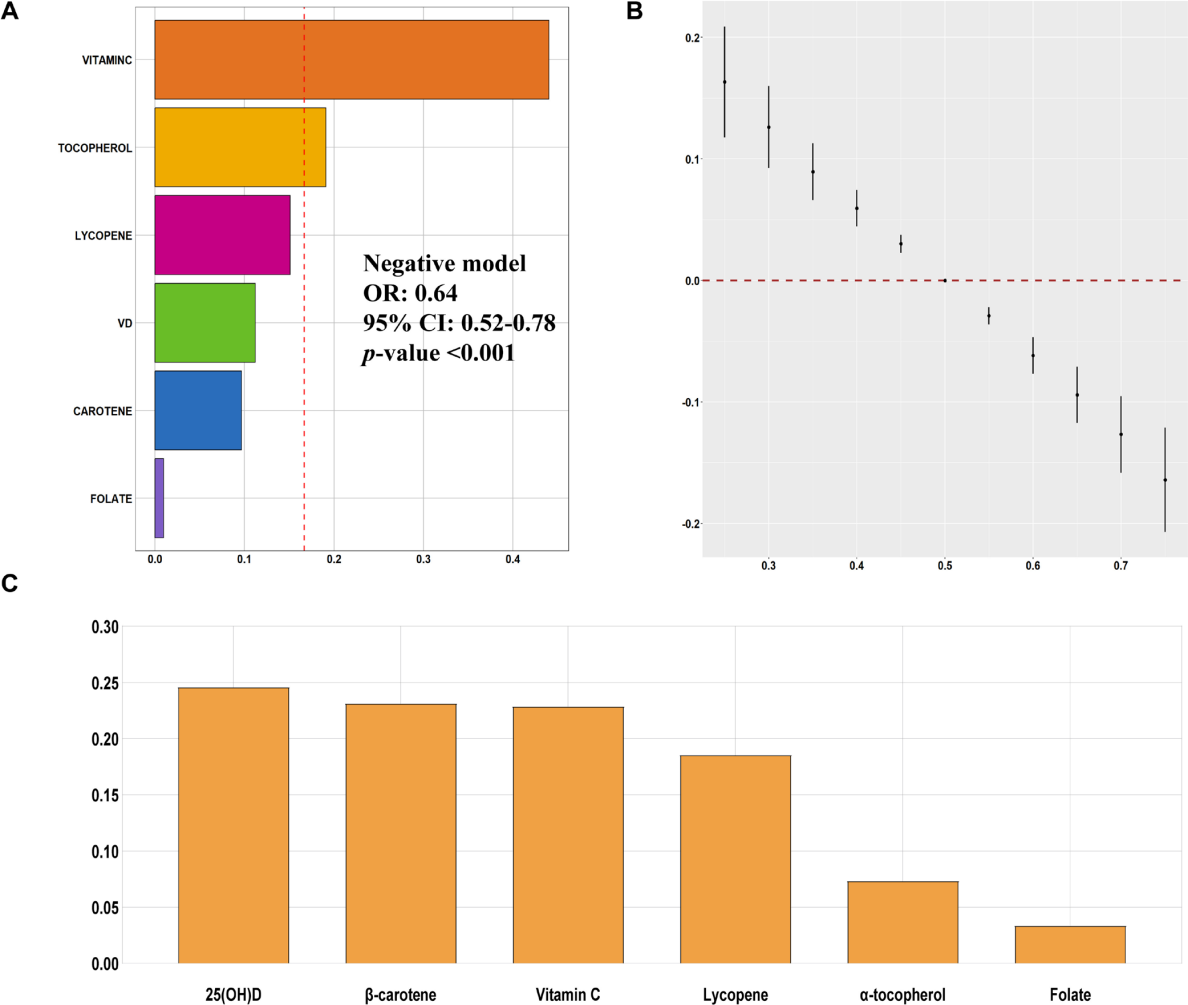
The association between serum micronutrients and advanced CKM syndrome. (A) The WQS model weights of serum micronutrients on the prevalence of advanced CKM syndrome in a negative direction. (B) Overall effect of serum micronutrient mixtures on the prevalence of advanced CKM syndrome in the BKMR model. (C) The Qgcomp model weights of serum micronutrients on the risk of advanced CKM syndrome.

The WQS index in the negative direction demonstrated that elevated serum micronutrient levels were negatively associated with the risk of advanced CKM syndrome (OR, 0.64; 95% CI: 0.52–0.78) (Figure 2A). While the WQS index for positive (Figure S3) did not show any significant relationship between the levels of micronutrients and the advanced CKM syndrome risk.

Figure 2B shows that compared to moderate levels of serum micronutrients, an increase in serum micronutrients above the 50th percentile is associated with a decrease in the incidence of advanced CKM syndrome. Table S8 also shows the summary of each PIP of six micronutrients.

Interestingly, the results of Qgcomp analysis also exhibited that the joint effect of all serum micronutrients in our study was negatively associated with the risk of advanced CKM syndrome (OR, 0.84; 95% CI: 0.79–0.89). Figure 2C presents that 25(OH)D received the highest weight of 0.2460 for advanced CKM syndrome risk, compared to weights of 0.2316, 0.2291, 0.1857, 0.0737, and 0.0339 for β‐carotene, vitamin C, lycopene, α‐tocopherol, and folate, respectively.

### 3.4 Feature Selection in ML Feature Selection, Evaluation, and Comparison of ML Models

Figure S4A shows the feature selection results based on the Boruta algorithm, in which age, folate, gender, lycopene, marital status, α‐tocopherol, PIR, β‐carotene, race and ethnicity, education, 25(OH)D, vitamin C, and smoking status were considered as key variables. The correlation matrix for the study variables is presented in Figure S4B. It is evident that some serum micronutrients exhibit correlations, such as vitamin C being significantly positively correlated with β‐carotene (*r* = 0.33) and α‐tocopherol (*r* = 0.31). As shown in Figure S4C, the results of the multicollinearity test confirm that there is no multicollinearity between variables. These features were subsequently integrated into our seven models. We used the original dataset to create a pairplot to illustrate the correlations among features. The pairplot uses a color‐coded system to distinguish between serum micronutrients, which aids in observing the correlations and distributions among features (Figure S5).

**FIGURE 3:**
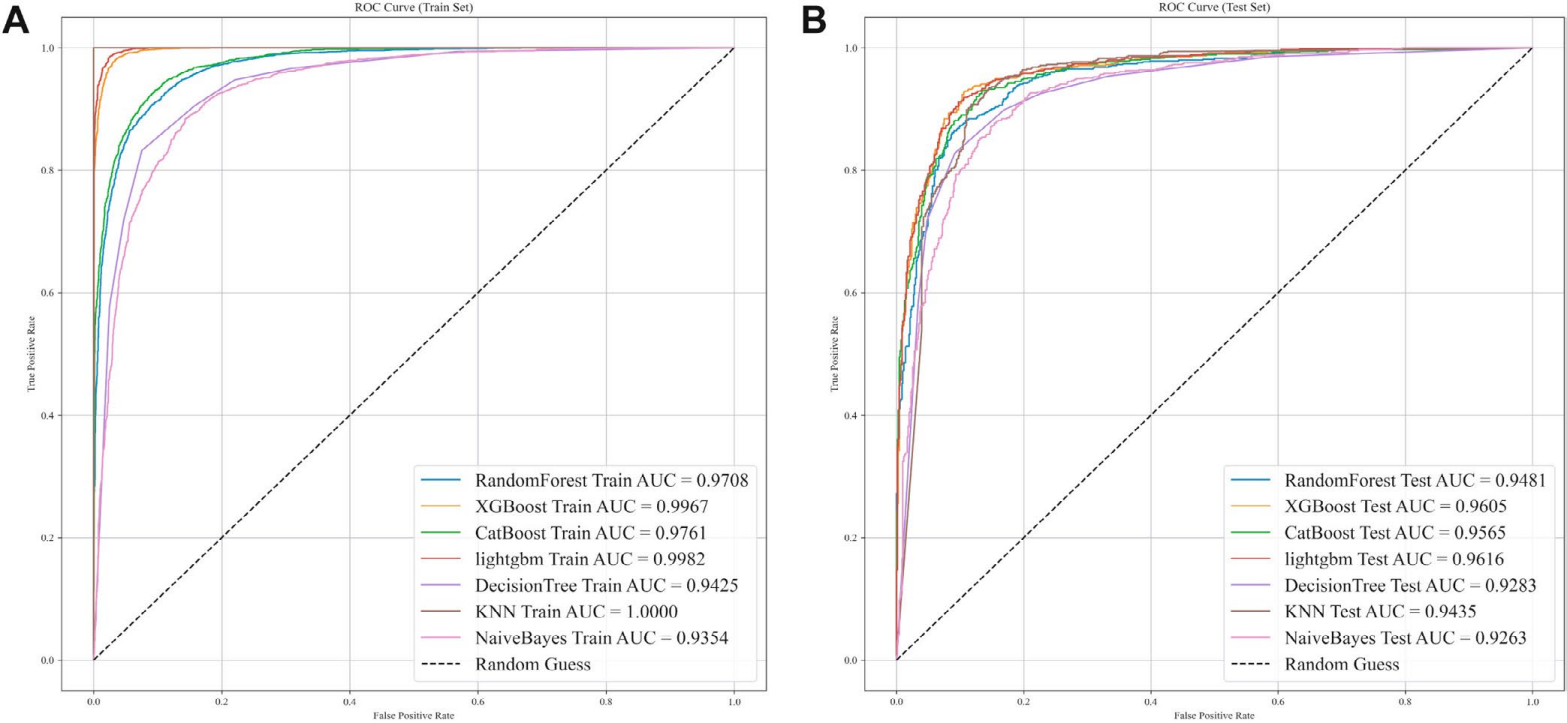
Performance comparison of machine learning models for advanced CKM syndrome prediction. (A) ROC curves for RF, XGB, CB, LGBM, DT, KNN, and NB in the training set, with AUC values shown in the legend. The dashed line represents the chance level (AUC = 0.5). (B) ROC curves for the same models in the testing set, with AUC values shown in the legend. The dashed line represents the chance level (AUC = 0.5). AUC, area under the curve; CB, CatBoost; DT, decision tree; KNN, k‐nearest neighbors; LGBM, LightGBM; NB, naïve Bayes; RF, random forest; XGB, XGBoost.

Then, we developed 7 prevalent ML models, including RF, XGB, CB, LGBM, DT, KNN, and NB to predict advanced CKM syndrome risk by serum micronutrients. Figure 3 shows the ROC curves for the training set and testing set of all models. In the training set, all models demonstrated high predictive performance, with LGBM (AUC = 0.998) and KNN (AUC = 1.000) achieving the highest scores, though KNN exhibited signs of overfitting. In the test set, LGBM (AUC = 0.962) maintained the best generalization performance, while KNN showed a decline in AUC (AUC = 0.944), suggesting reduced generalizability. To mitigate the bias caused by data imbalance, we carried out a comparative analysis of their evaluation indices, as presented in Table 3; the confusion matrix of the model is shown in Figure S6. All performance metrics were considered together; the LGBM model exhibited the best performance and model fit. These results suggested that LGBM is the most effective model for predicting advanced CKM syndrome risk based on serum nutrient profiles, providing a potential tool for early risk assessment and clinical decision‐making. Thus, the LGBM model was chosen for the subsequent analysis. In addition, the decision curve analysis (DCA) was conducted to evaluate the clinical practice (Figure S7).

**TABLE 3:** Comparison of the performance metrics among the 7 models.

Metrics	RF	XGB	CB	LGBM	DT	KNN	NB
AUC	0.948	0.96	0.956	0.962	0.928	0.944	0.926
95% CI	0.937, 0.959	0.951, 0.970	0.947, 0.966	0.942, 0.970	0.913, 0.942	0.931, 0.956	0.913, 0.939
Accuracy	0.879	0.909	0.892	0.904	0.869	0.871	0.856
Precision	0.873	0.896	0.886	0.888	0.898	0.808	0.852
Sensitive/Recall	0.884	0.923	0.897	0.921	0.828	0.971	0.858
Specificity	0.874	0.895	0.887	0.887	0.908	0.774	0.855
F1 score	0.879	0.91	0.892	0.904	0.862	0.882	0.855
FPR	0.126	0.105	0.113	0.113	0.092	0.226	0.145
FNR	0.116	0.077	0.103	0.079	0.172	0.029	0.142
PPV	0.873	0.896	0.886	0.888	0.898	0.808	0.852
NPV	0.886	0.923	0.898	0.919	0.844	0.965	0.861

Abbreviations: AUC, area under the curve; CB, CatBoost; DT, Decision Tree; FNR, false negative rate; FPR, false positive rate; KNN, k‐nearest neighbors; LGBM, LightGBM; NB, naïve Bayes; NPV, negative predictive value; PPV, positive predictive value; RF, random forest; XGB, XGBoost.

### 3.5 Model Interpretations With SHAP

**FIGURE 4:**
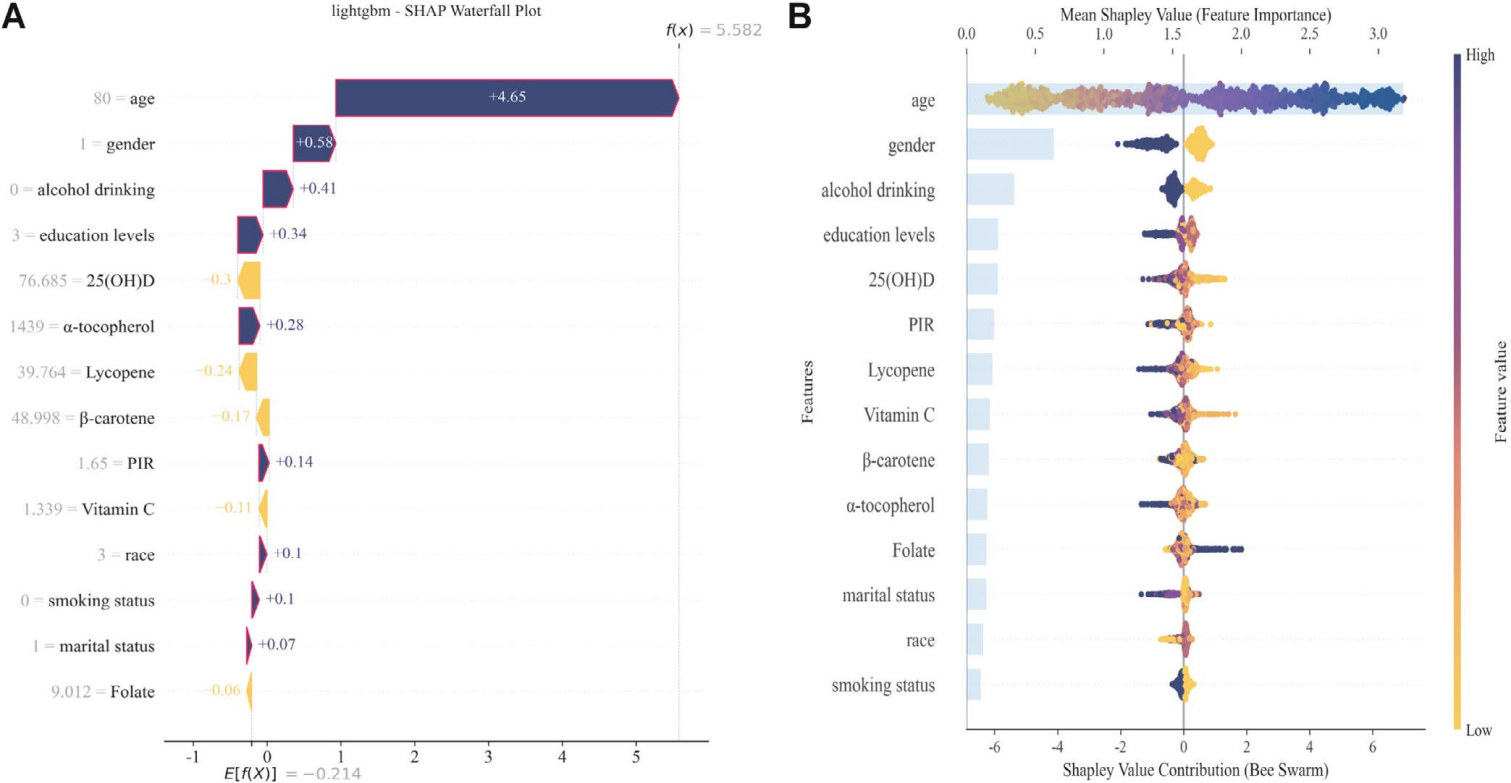
SHAP analysis of serum nutrient contributions to the risk of advanced CKM syndrome. (A) SHAP waterfall plot for the contribution of individual sample features to advanced CKM syndrome. The purple bar indicates the contribution of the variable to the development of advanced CKM syndrome in an individual, while the yellow bar represents its inhibitory effect. The *f*(*X*) values are probabilistic predictions, and *E*[*f*(*X*)] (the base value) represents the predictions made without any input to the model. *f*(*X*) corresponds to the log odds ratio for each observation. (B) The SHAP bar plot and bee swarm plot. Mean SHAP value ranking of the features in LGBM model; higher mean SHAP values correspond to a higher risk of advanced CKM syndrome. Each point represents a SHAP value for a feature and an instance, with the color indicating the feature value (purple = high, yellow = low). Features are ordered by importance, and the position along the *X*‐axis reflects the contribution to LGBM model's output.

In Figure 4, the SHAP plots for the LGBM model highlight key features associated with the risk of advanced CKM syndrome, offering valuable insights for developing personalized care strategies. For instance, for an 80‐year‐old female, regulating serum 25(OH)D levels may help reduce the risk of developing advanced CKM syndrome (Figure 4A). Figure 4B indicates that serum 25(OH)D and lycopene are the most significant predictors in the current model. Serum β‐carotene, α‐tocopherol, lycopene, vitamin C, and 25(OH)D are negatively associated with advanced CKM syndrome risk, whereas folate is positively associated. Additionally, we plotted the SHAP values in Table S9. And in Figure S8, the SHAP decision plot shows the cumulative contribution of different participants' features to their predicted risk.

### 3.6 The Independent and Synergistic Effects of Important Features on Prediction

**FIGURE 5:**
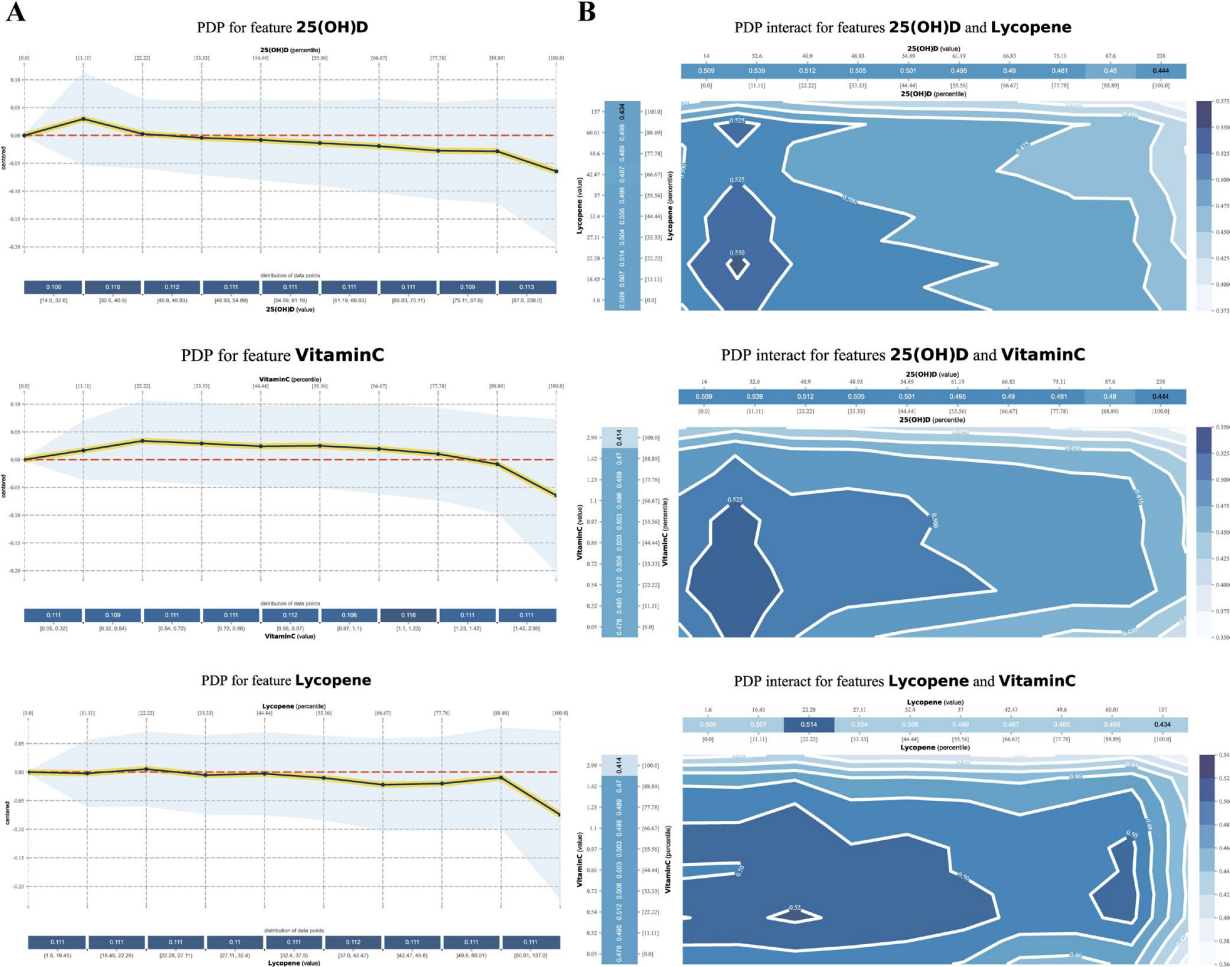
The independent and synergistic effects of serum micronutrients levels on the predicted risk of advanced CKM syndrome. (A) The PDPs illustrate the relationships between advanced CKM syndrome and 25(OH)D, vitamin C, and lycopene. The *x*‐axis represents the log‐transformed values of each serum micronutrients, while the *y*‐axis shows the change in the predicted advanced CKM syndrome risk. Shaded regions indicate confidence intervals, and the blue histograms below each plot visualize the distribution of data points across feature values. (B) Two‐dimensional partial dependence plots (2D PDPs) illustrating the interaction effects of serum micronutrients on the predicted risk of advanced CKM syndrome. Contour plots depict the combined influence of 25(OH)D and lycopene, 25(OH)D and vitamin C, and lycopene and vitamin C on advanced CKM syndrome risk. The *x*‐axis and *y*‐axis represent the log‐transformed values of the two serum micronutrients, with each grid corresponding to a 10% range of their levels. The contour plot shows the impact of these micronutrients on the predicted advanced CKM syndrome outcome.

We use PDPs to gain a more comprehensive understanding of how individual characteristics affect the risk prediction of advanced CKM syndrome. Figure 5A shows the relationship between six serum micronutrients and their impact on the risk of advanced CKM syndrome in the LGBM model. Analysis of PDPs suggests that vitamin C and 25(OH)D may have protective effects, and higher levels may reduce the risk of advanced CKM syndrome. Moreover, β‐carotene may have a protective effect at low concentrations, but its effect tends to stabilize or slightly increase at high concentrations, suggesting a possible dose‐dependent effect. In addition, the effects of α‐tocopherol and lycopene on the risk of advanced CKM syndrome are relatively small and may not be the main determining factors. We found that the protective effects of vitamin C and 25(OH)D on advanced CKM syndrome tend to saturate after reaching a certain concentration, which may suggest that excessive supplementation may not bring additional health benefits. Figure S9 displays the PDPs for the remaining three serum micronutrients included in the constructed ML model.

Next, we also conducted PDPs binary analysis to investigate the interaction between two serum micronutrients in predicting advanced CKM syndrome. Figure 5B depicts the combined influence of serum 25(OH)D and lycopene levels on the risk of advanced CKM syndrome. It reveals that 25(OH)D serves as a significant protective factor against advanced CKM risk. Additionally, the protective effects of other nutrients, such as lycopene and vitamin C, may be diminished when 25(OH)D levels are high. Figure 5B also illustrates the synergistic effects on the levels of 25(OH)D and vitamin C in serum, revealing that vitamin C and lycopene may have a synergistic effect, meaning that when both are low, the risk of advanced CKM is highest, while when at least one is high, the risk decreases. These results supported a nutritional combination intervention strategy, optimizing the intake of 25(OH)D, vitamin C, and lycopene, which may help reduce the risk of advanced CKM syndrome.

## 4 Discussion

Based on the big data from the NHANES, we could conclude that six serum micronutrients are negatively associated with advanced CKM syndrome prevalence, as evidenced by traditional multivariable regression models and mixture effect analysis models. Among the seven ML models considered in our study, the LGBM model performed the best. The average AUC of the LGBM model is 0.962, indicating excellent efficiency and stability in classification. Using the SHAP method, we determined that serum levels of 25(OH)D and lycopene are the most important factors in predicting advanced CKM syndrome. Controlling these variables can develop personalized care plans. PDPs indicate that at specific levels, serum 25(OH)D and vitamin C are negatively correlated with the risk of advanced CKM syndrome. These results suggest that serum 25(OH)D, lycopene, and vitamin C are key predictive factors for advanced CKM syndrome. To our knowledge, this is the first study to develop and validate a predictive model for the correlation between serum micronutrients and advanced CKM syndrome, which combines serum micronutrients with baseline features. In addition, we used ML models LGBM and SHAP analysis frameworks to obtain relatively more reliable results.

Previous studies suggested that the levels of serum micronutrients, such as 25(OH)D and vitamin C, are associated with CVD (Tian et al. 2022; Wang et al. 2012). Li (2023) reported that low vitamin C status was associated with a higher risk of CKD. Additionally, a recent investigation demonstrated that elevated serum lycopene levels are significantly associated with a reduced risk of mortality from all causes and CVD among patients with CKD (Zhong et al. 2022). However, the association between serum micronutrients and advanced CKM syndrome is still unclear. Our findings provide stronger evidence supporting the pivotal role of serum micronutrients in the pathogenesis of advanced CKM syndrome. This study found that the effects of serum folate on advanced CKM syndrome may differ due to varying covariate adjustment strategies. This may be related to population‐specific factors, such as vitamin B12 deficiency.

Vitamin C, β‐carotene, α‐tocopherol, and lycopene are some micronutrients that function as antioxidants. They can neutralize free radicals in cells, thereby reducing oxidative damage and inflammation (Gombart et al. 2020). The potential mechanism linking these serum micronutrients to the risk of advanced CKM syndrome may involve oxidative stress and inflammation. Inflammation can secrete pro‐inflammatory cytokines such as tumor necrosis factor‐α (TNF‐α) and interleukin‐6 (IL‐6) by regulating various signaling pathways, including vascular endothelial growth factor (VEGF), nuclear factor‐kappa B (NF‐κB), and transforming growth factor beta (TGF‐β). These processes can contribute to atherosclerosis, impair endothelial function, and increase vascular rigidity, ultimately leading to hypertension and heart failure (Cao et al. 2025; Ndumele, Rangaswami, et al. 2023; Neeland et al. 2019). In kidney disease, inflammation leads to kidney damage through glomerular injury and fibrosis, resulting in the progression of CKD (Brennan et al. 2021; Speer et al. 2022; Wu et al. 2022). Metabolically, inflammation is related to insulin resistance. Insulin resistance is a key feature of type 2 diabetes and MetS, which aggravates visceral fat storage and further inflammation (Glass and Olefsky 2012).

Several potential mechanisms may explain how 25(OH)D deficiency contributes to advanced CKM syndrome. These mechanisms include inflammatory pathways, the regulation of nitric oxide, oxidative stress, and the activation of the renin‐angiotensin‐aldosterone system (Renke et al. 2023). 25(OH)D can significantly reduce endothelial dysfunction and damage caused by oxidative stress, while also regulating the transcription of endothelial NO synthase. Additionally, 25(OH)D deficiency is notably associated with oxidative stress, inflammation, and major chronic diseases related to aging. Impaired signaling due to 25(OH)D deficiency can contribute to hypertension. Furthermore, 25(OH)D provides anti‐inflammatory and anti‐foam cell benefits.

Our study has some strengths. Firstly, our study used multiple models including multivariate regression analysis, WQS, QGcomp, and BKMR regression analysis to validate the association between serum micronutrients and advanced CKM syndrome. Multiple models analyzed data from different perspectives such as linear/nonlinear, individual/mixed effects, and parametric/nonparametric, forming a multidimensional evidence chain to enhance the comprehensiveness of the analysis and the robustness of the conclusions. Secondly, compared to previous studies, the innovation of this study lies in its methodological approach. We applied 7 different models to the dataset and concluded that LGBM outperformed the others based on several performance indicators. By leveraging the high‐precision predictive capabilities of the LGBM model (AUC = 0.962) alongside SHAP interpretability analysis, this study not only validates established risk factors such as old age and sex but also identifies the independent contribution of 25(OH)D and lycopene.

However, there are also potential limitations. To establish causal relationships, cross‐sectional data is inadequate, and longitudinal studies in the future will enhance our ML models. Furthermore, there may also be a reverse causal relationship between CKM syndrome and serum micronutrient levels. For example, advanced CKM syndrome may lead to malabsorption of nutrients. The constructed ML model lacks external data validation. In the future, we need to use the model in this study as a basis and conduct multi‐center external validation using data from large public or private databases. Besides, we should evaluate the relationship between serum micronutrient levels measured at baseline and advanced CKM syndrome. A single measurement cannot capture the temporal changes in serum micronutrient levels, potentially leading to misclassification of individual micronutrient exposure levels. Measurements across different periods may be subject to batch effects. Additionally, the study relies solely on complete‐case analysis and excludes individuals with missing data, which may introduce selection bias. Notably, the study population is restricted to the local population in the United States, and no self‐built cohort has been used to validate findings in other population databases. This limitation may hinder the generalizability of the results and conclusions to other countries and regions.

## 5 Conclusion

To conclude, this study identified a significant relationship between serum micronutrients and the risk of advanced CKM syndrome in the US population, emphasizing the crucial role of micronutrients in disease development. Utilizing a robust ML model and SHAP analysis, we identified vitamin C, 25(OH)D, and lycopene as key predictors, providing guidance for the development of personalized clinical care strategies and supporting the development of precise nutritional supplementation plans.

## Author Contributions

**Jingbo Zhang:** conceptualization, methodology, formal analysis, visualization, investigation, resources, writing, supervision, validation, project administration, funding acquisition. **Yi Ou:** conceptualization, methodology, visualization, writing, validation. **Genlong Bai:** writing, formal analysis, investigation, validation. **Yidian Fu:** writing, validation. **Xiang Qu:** writing, validation. **Qian Liu:** investigation, validation. **Jin Chen:** supervision, funding acquisition, project administration. **Xinyi Shao:** visualization, investigation, supervision, validation, funding acquisition. **Yuan Zhan:** visualization, investigation, supervision, validation. **Aijun Chen:** validation, project administration, writing, funding acquisition.

## Ethics Statement

The survey plan and study procedures received approval from the NCHS Ethics Review Board As this study involved secondary data analysis from NHANES, it was exempt from institutional review.

## Consent

Written informed consent was collected from all participants.

## Conflicts of Interest

The authors declare no conflicts of interest.

## Supporting information


**Figure S1:** Flowchart of the participant selection.
**Figure S2:** Oversampling of the participant.
**Figure S3:** The WQS model weights of serum micronutrients on the prevalence of advanced CKM syndrome in positive direction.
**Figure S4:** Feature selection, correlation analysis, and multicollinearity assessment of predictor variables. (A) Feature importance ridge plot based on the Boruta algorithm. The Boruta method was applied to identify the most relevant features for the predictive model. Orange‐colored features were confirmed as important, while gray‐colored features were rejected. (B) Feature correlation heatmap. Pearson correlation coefficients were calculated to evaluate the relationships between predictor variables. The color gradient represents the strength and direction of correlations, with dark blue indicating strong negative correlations and dark yellow indicating strong positive correlations. (C) Variance Inflation Factor (VIF) analysis. VIF values were computed to assess multicollinearity among predictor variables. All VIF values were below 2, indicating minimal collinearity concerns.
**Figure S5:** Pairplot of six serum micronutrients.
**Figure S6:** The confusion matrix of the models.
**Figure S7:** The decision curve analysis curves reflect the net benefit of different models for advanced CKM syndrome.
**Figure S8:** The SHAP decision plot. Features are arranged along the y‐axis based on the mean of their absolute SHAP values. A feature's position higher in the plot indicates greater importance to the model. The red line signifies that the individual was predicted to be associated with increased advanced CKM risk, whereas the blue line indicates a state of good health.
**Figure S9:** Relationships between α‐tocopherol, β‐carotene, folate and advanced CKM syndrome.
**Table S1:** Definitions of CKM conditions.
**Table S2:** Methods for evaluating each CKM stage.
**Table S3:** Detailed algorithm of the simplified 10‐year CVD risk models.
**Table S4:** Assessment of multicollinearity of features using variance inflation factors (VIF).
**Table S5:** Hyperparameter for machine learning models.
**Table S6:** The calculation formulas for the machine learning model metrics.
**Table S7:** Confusion matrix for classification of advanced CKM.
**Table S8:** PIPs of each serum micronutrients for the prevalence of advanced CKM syndrome in BKMR model.
**Table S9:** Mean SHAP values.

## Data Availability

The all datasets used for analyses are publicly available at CDC NHANES (https://www.cdc.gov/nchs/nhanes/index.htm). The code will be made available upon request.
