# Supplementary material for: Association Between Serum Micronutrients and Advanced CKM Syndrome: An Interpretable Machine Learning‐Based Study
Source: Food Sci Nutr. 2025 Oct 22;13(10):e71059. doi: 10.1002/fsn3.71059 (PMC12541883; doi:10.1002/fsn3.71059)
Supplement: Supplementary file 1 — Figure S1: Flowchart of the participant selection. Figure S2: Oversampling of the participant. Figure S3: The WQS model weights of serum micronutrients on the prevalence of advanced CKM syndrome in positive direction. Figure S4: Feature selection, correlation analysis, and multicollinearity assessment of predictor variables. (A) Feature importance ridge plot based on the Boruta algorithm. The Boruta method was applied to identify the most relevant features for the predictive model. Orange‐colored features were confirmed as important, while gray‐colored features were rejected. (B) Feature correlation heatmap. Pearson correlation coefficients were calculated to evaluate the relationships between predictor variables. The color gradient represents the strength and direction of correlations, with dark blue indicating strong negative correlations and dark yellow indicating strong positive correlations. (C) Variance Inflation Factor (VIF) analysis. VIF values were computed to assess multicollinearity among predictor variables. All VIF values were below 2, indicating minimal collinearity concerns. Figure S5: Pairplot of six serum micronutrients. Figure S6: The confusion matrix of the models. Figure S7: The decision curve analysis curves reflect the net benefit of different models for advanced CKM syndrome. Figure S8: The SHAP decision plot. Features are arranged along the y‐axis based on the mean of their absolute SHAP values. A feature's position higher in the plot indicates greater importance to the model. The red line signifies that the individual was predicted to be associated with increased advanced CKM risk, whereas the blue line indicates a state of good health. Figure S9: Relationships between α‐tocopherol, β‐carotene, folate and advanced CKM syndrome. Table S1: Definitions of CKM conditions. Table S2: Methods for evaluating each CKM stage. Table S3: Detailed algorithm of the simplified 10‐year CVD risk models. Table S4: Assessment of multicollinearity of features u [file FSN3-13-e71059-s001.docx]

**Supplementary materials**


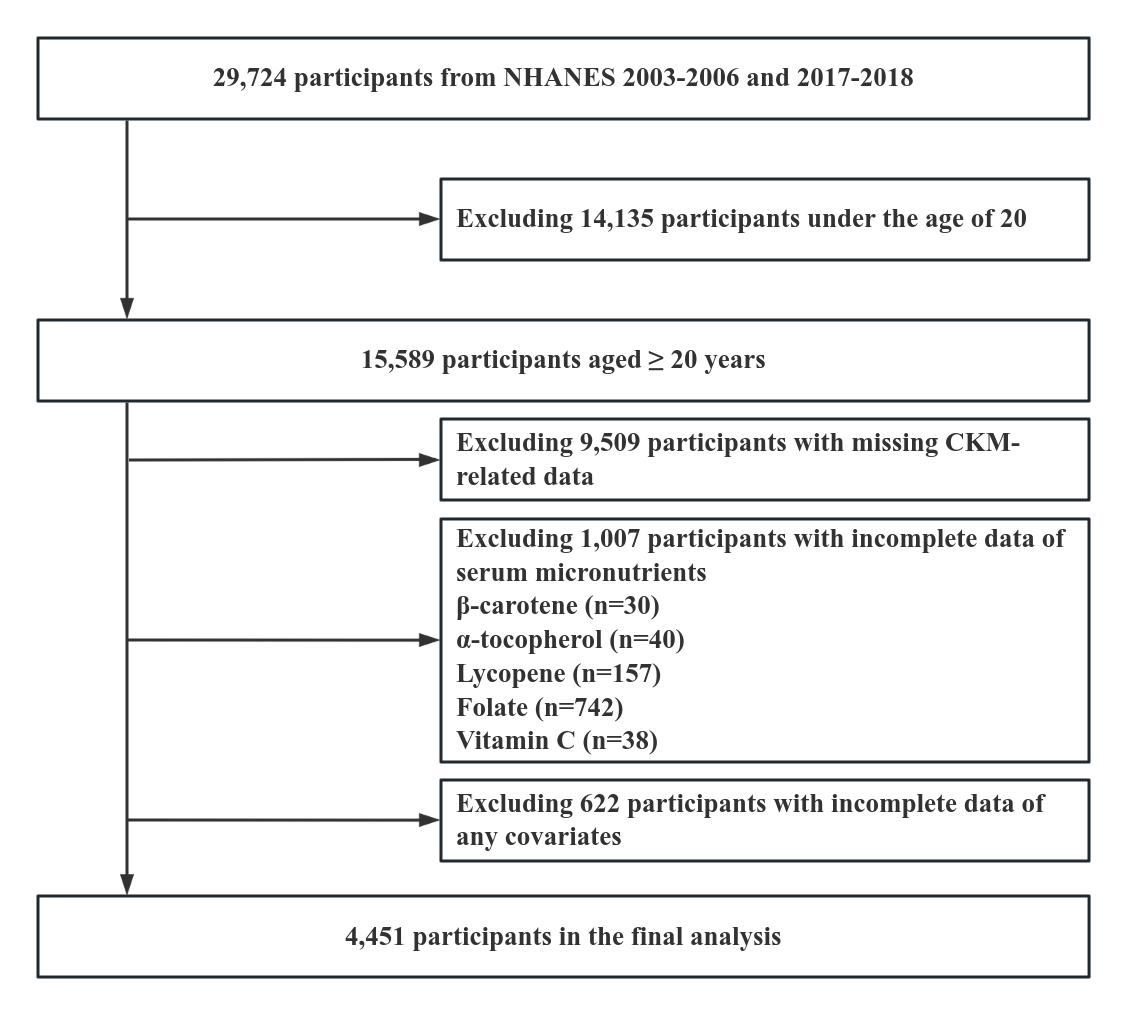


**Figure S1. Flowchart of the participant selection.**


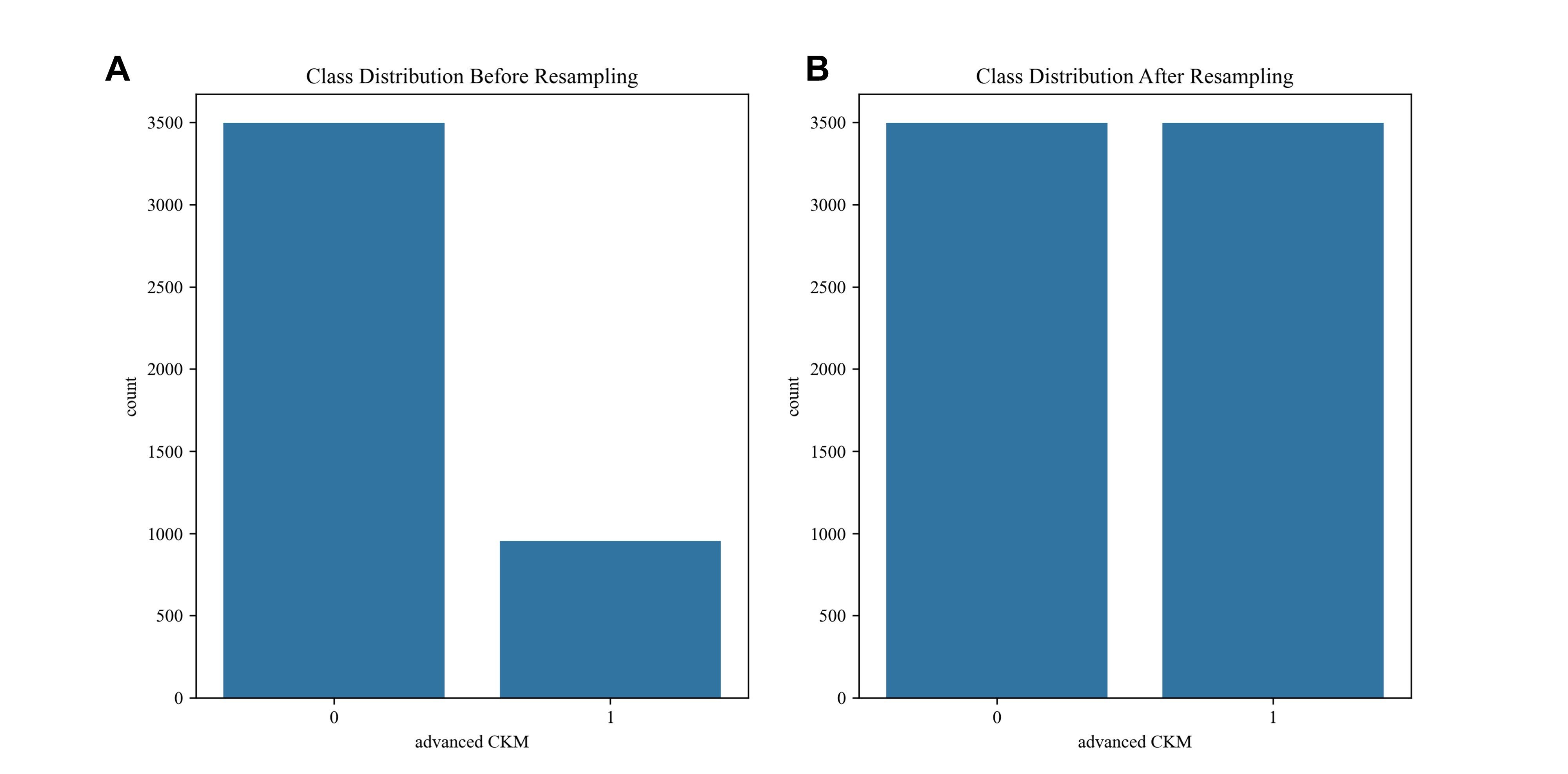


**Figure S2. Oversampling of the participant.**


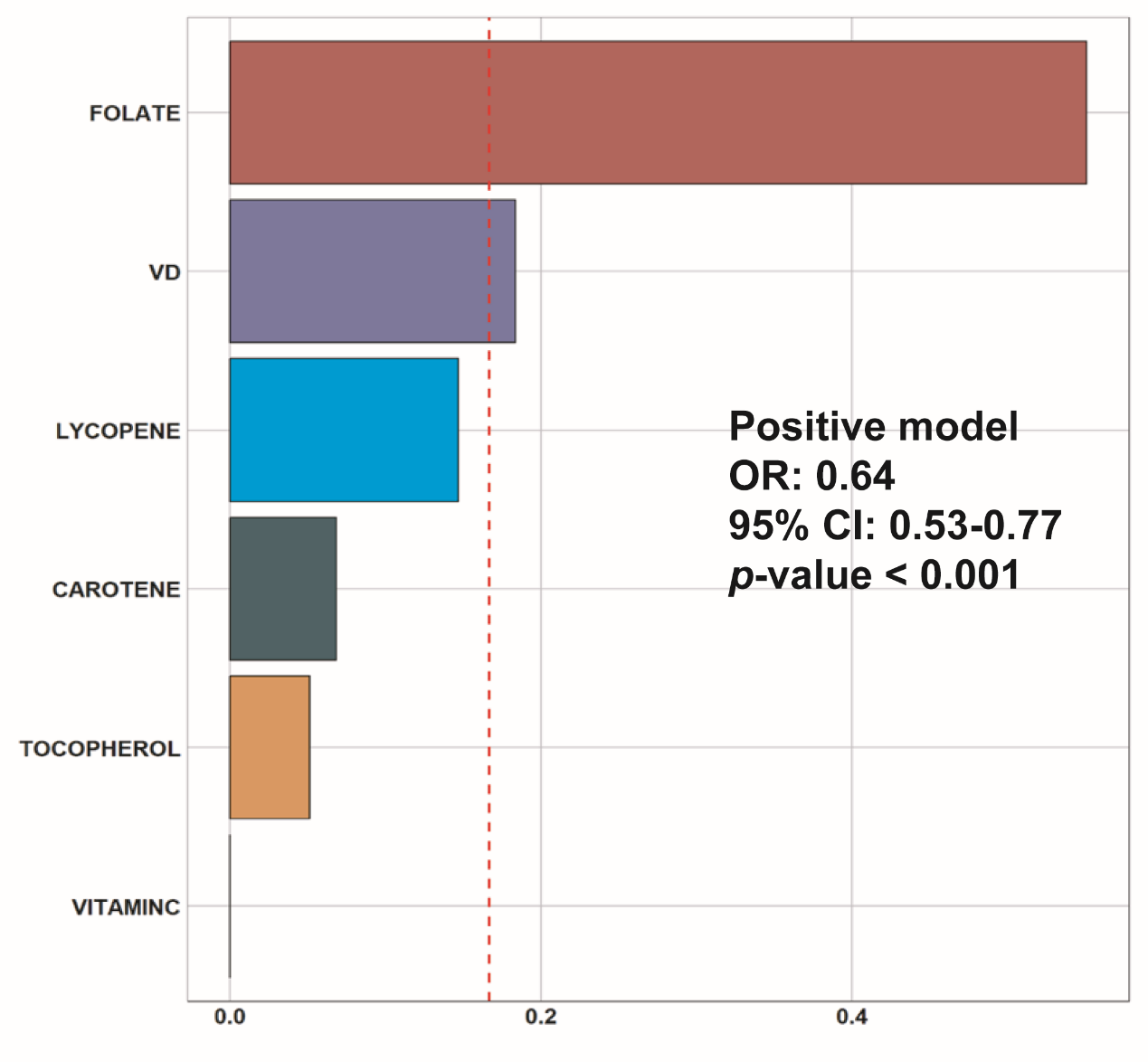


**Figure S3. The WQS model weights of serum micronutrients on the prevalence of advanced CKM syndrome in positive direction.**


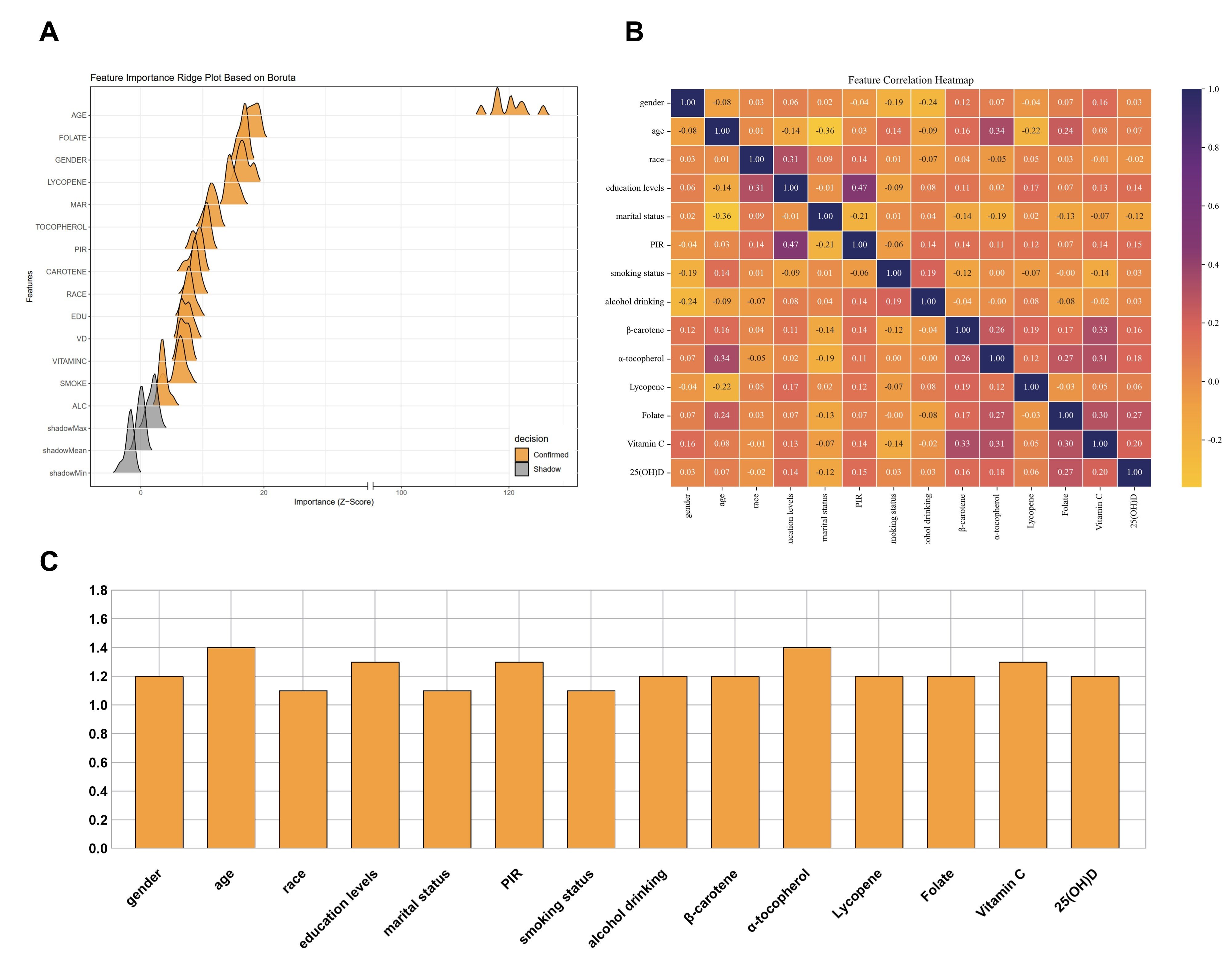


**Figure S4. Feature selection, correlation analysis, and multicollinearity assessment of predictor variables.** (A) Feature importance ridge plot based on the Boruta algorithm. The Boruta method was applied to identify the most relevant features for the predictive model. Orange-colored features were confirmed as important, while gray-colored features were rejected. (B) Feature correlation heatmap. Pearson correlation coefficients were calculated to evaluate the relationships between predictor variables. The color gradient represents the strength and direction of correlations, with dark blue indicating strong negative correlations and dark yellow indicating strong positive correlations. (C) Variance Inflation Factor (VIF) analysis. VIF values were computed to assess multicollinearity among predictor variables. All VIF values were below 2, indicating minimal collinearity concerns.


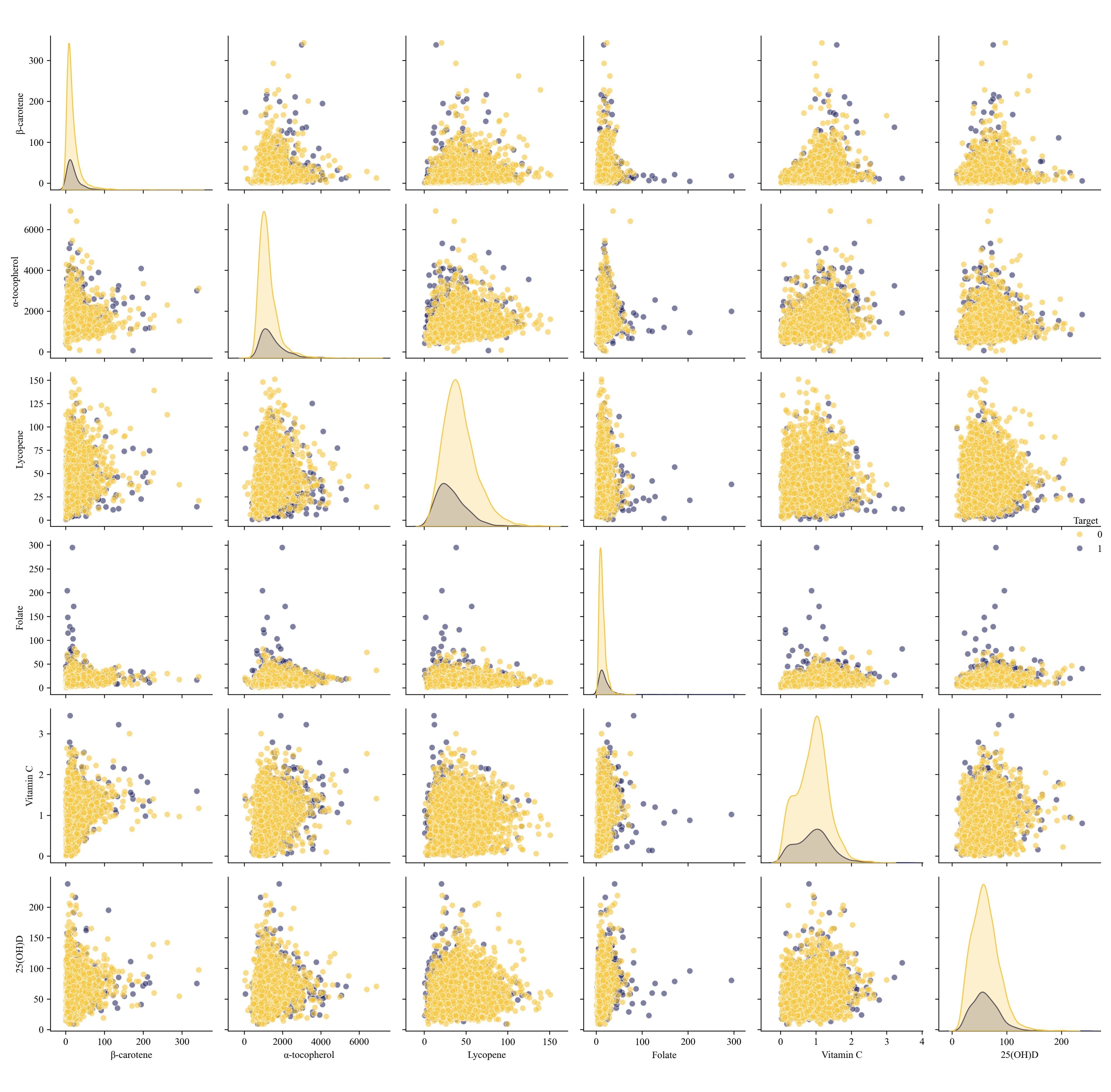


**Figure S5. Pairplot of six serum micronutrients.**


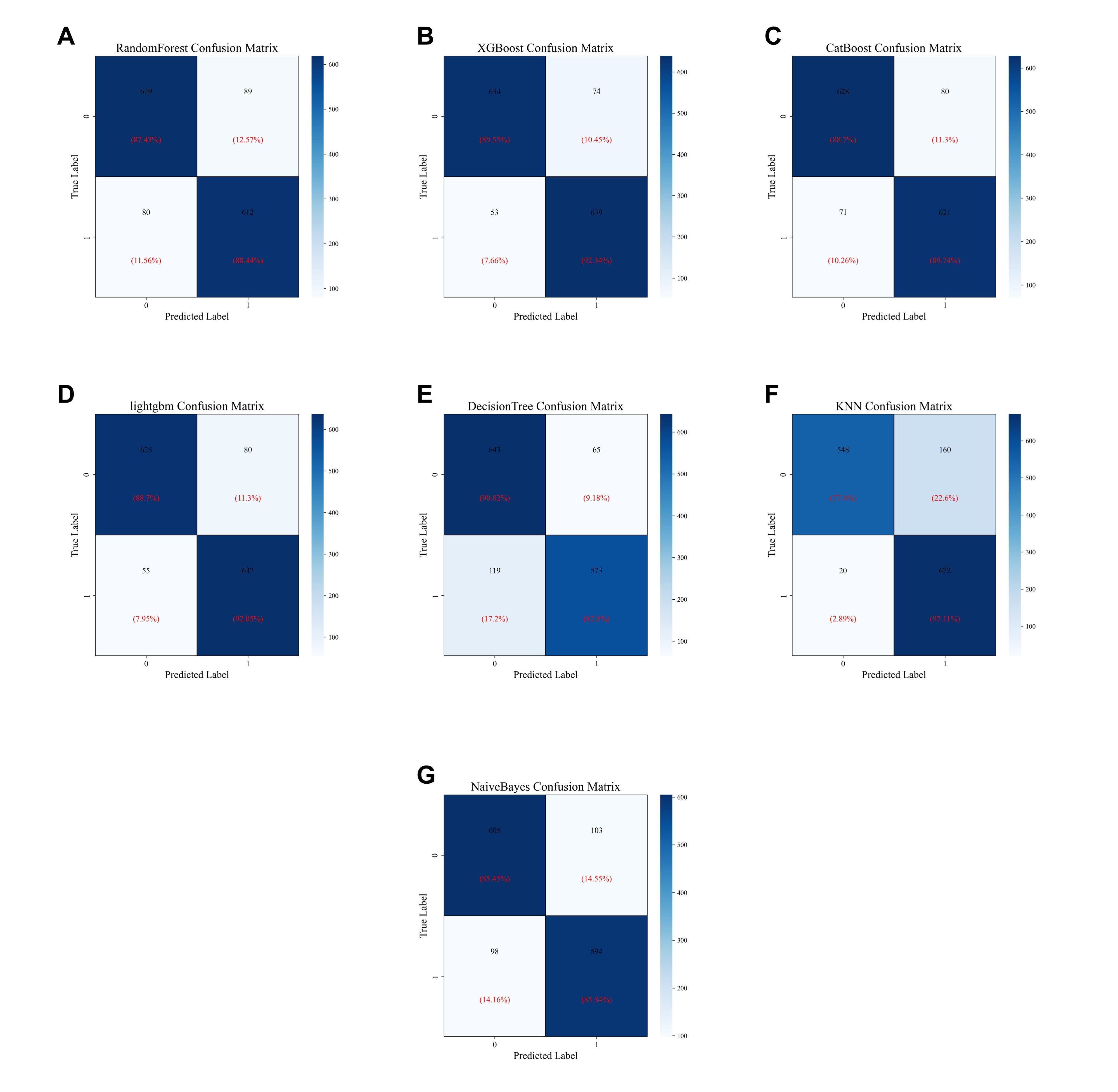


**Figure S6. The confusion matrix of the models.**


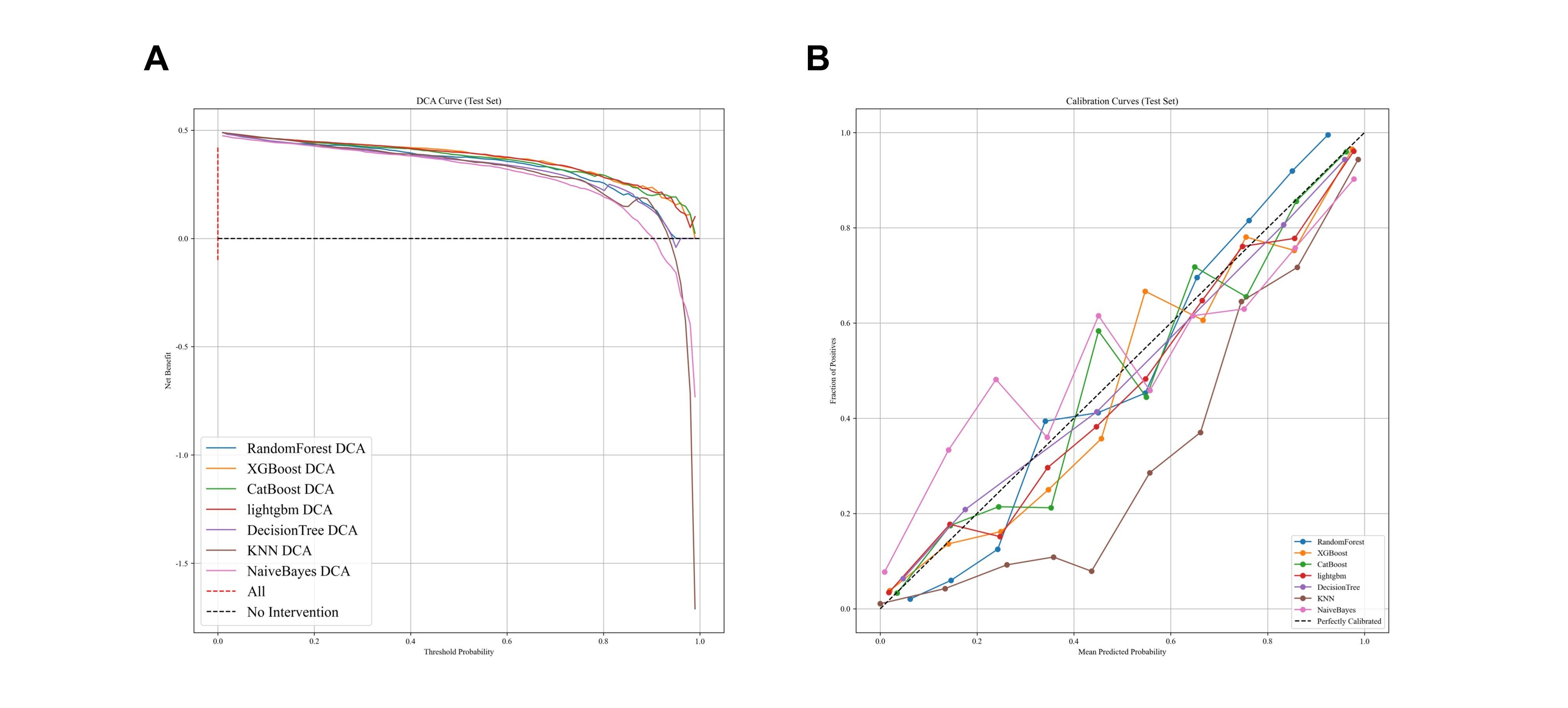


**Figure S7. The decision curve analysis curves reflect the net benefit of different models for advanced CKM syndrome.**


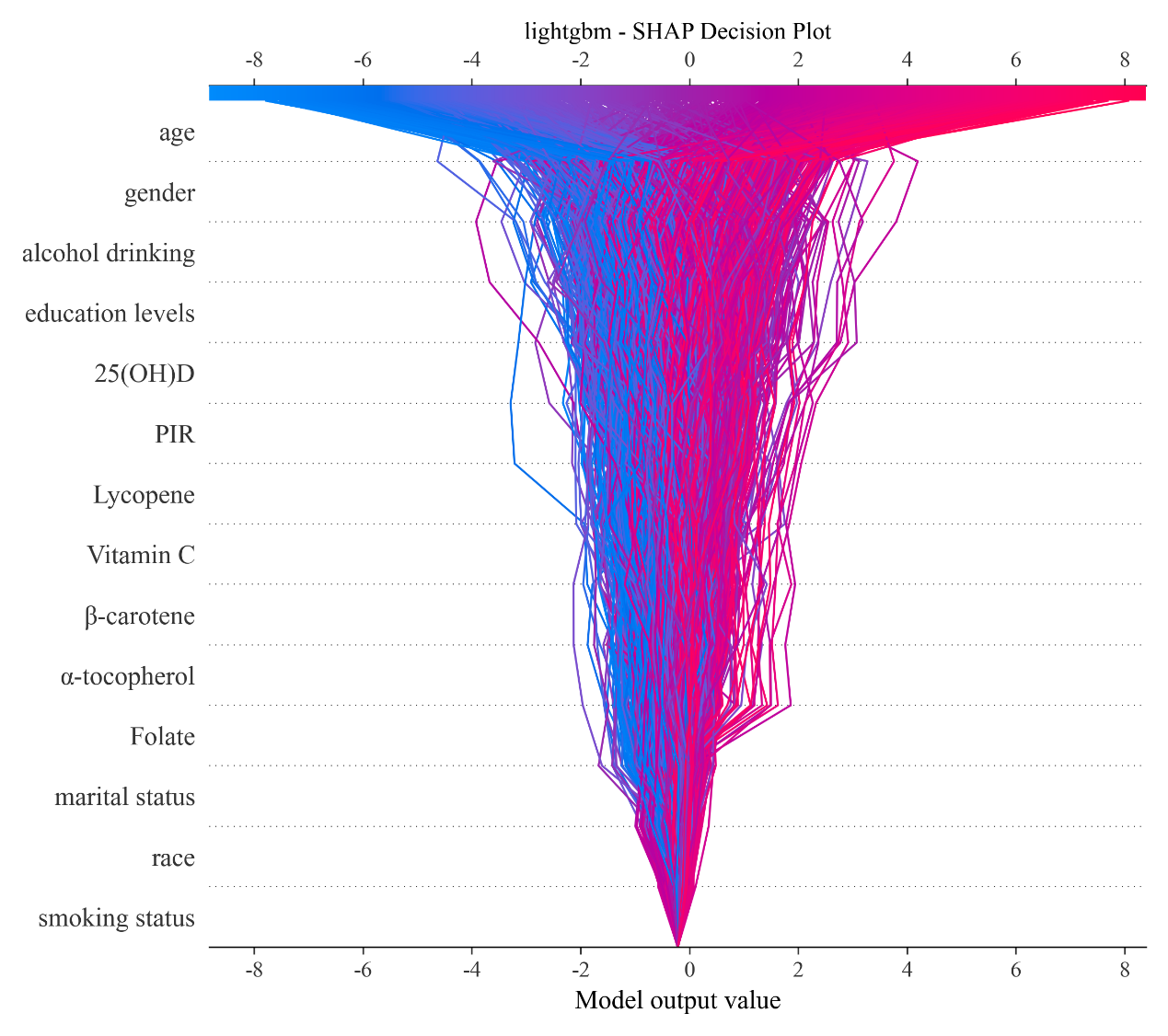


**Figure S8. The SHAP decision plot. Features are arranged along the y-axis based on the mean of their absolute SHAP values.** A feature’s position higher in the plot indicates greater importance to the model. The red line signifies that the individual was predicted to be associated with increased advanced CKM risk, whereas the blue line indicates a state of good health.


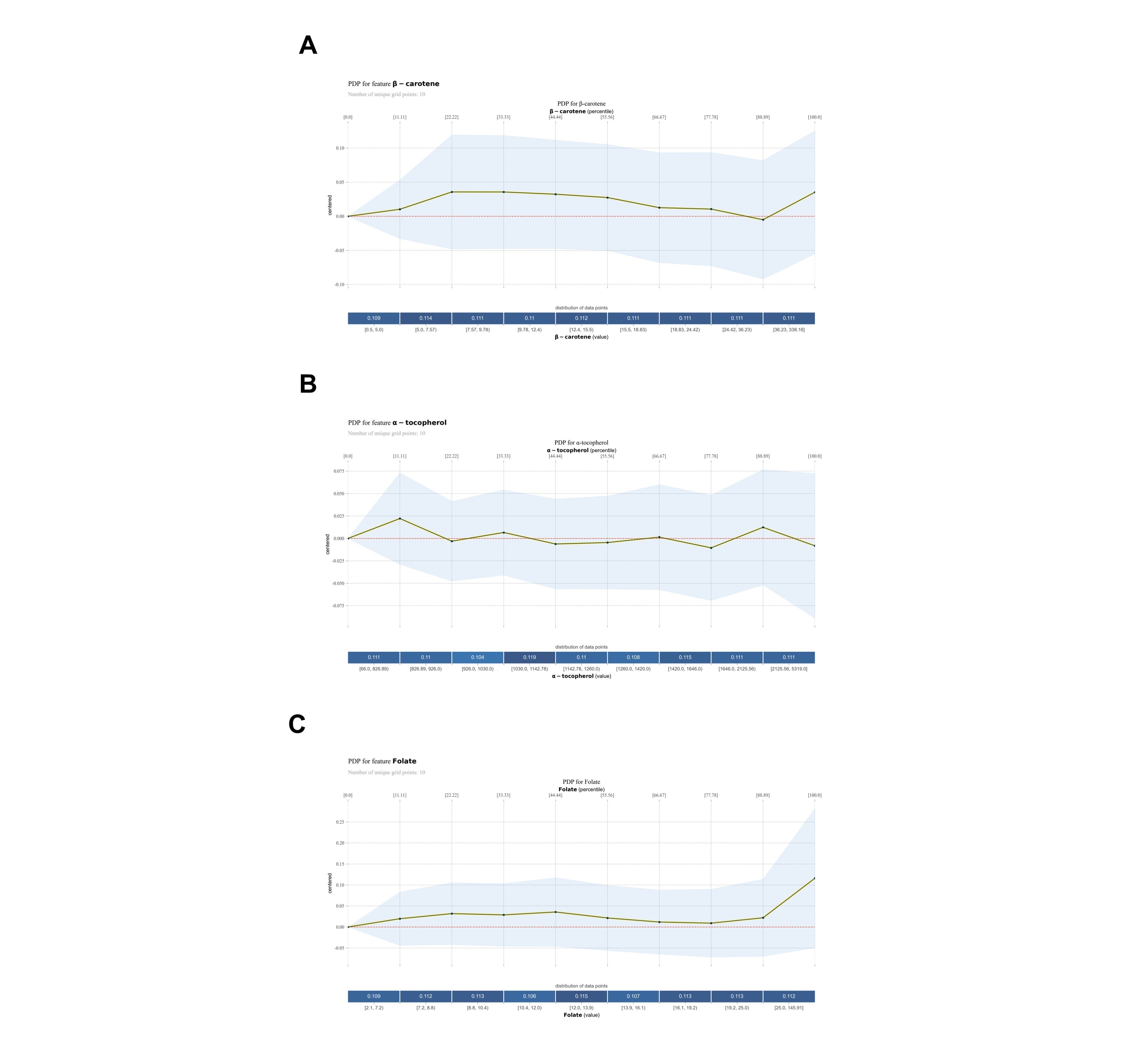


**Figure S9. Relationships between α-tocopherol, β-carotene, folate and advanced CKM syndrome.**

**Supplementary Table 1. Definitions of CKM conditions**

| **CKM**  **conditions** | **Definition** | **CKM indicators** | **Threshold for CKM indicators** |
| --- | --- | --- | --- |
| CVD | Individuals with clinical CVD or subclinical CVD | Clinical CVD | History of chronic heart failure, coronary heart disease, heart attack, or stroke |
|  |  | Subclinical CVD | Any of the following criterion is met:  1) Very high-risk CKD in KDIGO classification: UACR≥300 mg/g and eGFR≤45-59 ml/min/1.73m², UACR≥30 mg/g and eGFR≤30-44 ml/min/1.73m², or eGFR≤29 ml/min/1.73m²  2) Predicted 10-year CVD risk≥20% |
| Kidney diseases | Individuals with CKD | CKD | Moderate-to-high-risk CKD in KDIGO  classification: UACR≥30 mg/g and eGFR≥60 ml/min/1.73m², UACR<300 mg/g and eGFR≤45-59 ml/min/1.73m², or UACR<30 mg/g and eGFR≤30-44 ml/min/1.73m² |
| Metabolic disorders | Individuals with overweight/obesity, abdominal obesity, prediabetes, diabetes, hypertension, hypertriglyceridemia or MetS | Overweight/obesity | BMI≥25 kg/m² (or ≥23 kg/m² if Asian ancestry) * |
|  |  | Abdominal obesity | Waist circumference ≥88/102 cm in female/male (or if Asian ancestry≥80/90 cm in female/male) |
|  |  | Prediabetes | Fasting blood glucose≥100-124 mg/dL or HbA1c≥5.7%-6.4% and without self-reported diagnosis of diabetes, use of insulin, or oral hypoglycemic agents |
|  |  | Diabetes | Fasting blood glucose ≥125 mg/dL or HbA1c≥6.5% or self-reported diagnosis of diabetes, use of insulin, or oral hypoglycemic agents |
|  |  | Hypertension | SBP≥130 mmHg or DBP≥80 mmHg or self-reported diagnosis of hypertension or use of antihypertensive medications |
|  |  | Hypertriglyceridemia | Triglycerides ≥135 mg/dL |
|  |  | MetS | MetS is defined by the presence of 3 or more of the following: |

**Supplementary Table 2. Methods for evaluating each CKM stage.**

| **CKM stages** | **Definition** | **Criterion** | **Threshold for CKM conditions** |
| --- | --- | --- | --- |
| Stage 0:  No CKM risk factors | Individuals with normal BMI and waist circumference, normoglycemia, normotension, a normal lipid profile, and no evidence of CKD or subclinical or clinical CVD | All criteria are met | BMI<25 kg/m² (or <23 kg/m²if Asian ancestry)* |
|  |  |  | Waist circumference <88/102 cm in female/male (or if Asian ancestry <80/90 cm in female/male) |
|  |  |  | Fasting blood glucose<100 mg/dL and HbA1c<5.7%and without self-reported diagnosis of diabetes, use of insulin or oral hypoglycemic agents |
|  |  |  | SBP<130 mm Hg and DBP<80 mm Hg without self-reported diagnosis of hypertension or use of antihypertensive medications |
|  |  |  | HDL cholesterol <50/40 mg/dL in female/male and triglycerides<150 mg/dL |
|  |  |  | Low-risk CKD in KDIGO classification according to eGFR and UACR: UACR<30 mg/g and eGFR≥60 ml/min/1.73m². |
|  |  |  | Predicted 10-year CVD risk<20% |
|  |  |  | No clinical CVD |
| Stage 1:  Excess or dysfunctional adiposity | Individuals with overweight/obesity, abdominal obesity, or dysfunctional adipose tissue, without the presence of other metabolic risk factors or CKD | Any of the three criteria is met | Overweight/obesity |
|  |  |  | Abdominal obesity |
|  |  |  | Prediabetes |
|  |  | All criteria are met | SBP<130 mm Hg and DBP<80 mm Hg without self-reported diagnosis of hypertension or use of antihypertensive medications |
|  |  |  | HDL cholesterol <50/40 mg/dL in female/male and triglycerides <150 mg/dL |
|  |  |  | Low-risk CKD in KDIGO classification according to eGFR and UACR: UACR<30 mg/g and eGFR≥60 ml/min/1.73m². |
|  |  |  | Predicted 10-year CVD risk<20% |
|  |  |  | No clinical CVD |
| Stage 2:  Metabolic risk factors and CKD | Individuals with metabolic risk factors (hypertriglyceridemia, hypertension, MetS, diabetes), or CKD | Any of the five criteria is met | Hypertriglyceridemia |
|  |  |  | Hypertension |
|  |  |  | diabetes |
|  |  |  | MetS |
|  |  |  | Moderate-to-high-risk CKD in KDIGO classification |
|  |  | All criteria are met | No very high-risk CKD in KDIGO classification |
|  |  |  | Predicted 10-year CVD risk<20% |
|  |  |  | No clinical CVD |
| Stage 3:  Subclinical CVD in CKM | Subclinical CVD among individuals with excess/dysfunctional adiposity, other metabolic risk factors, or CKD | Any of the two criteria is met | Very high-risk CKD in KDIGO classification |
|  |  |  | Predicted 10-year CVD risk≥20% |
|  |  | Any of the eight criteria is met | Overweight/obesity |
|  |  |  | Abdominal obesity |
|  |  |  | Prediabetes |
|  |  |  | Hypertriglyceridemia |
|  |  |  | Hypertension |
|  |  |  | diabetes |
|  |  |  | MetS |
|  |  |  | Moderate-to-high-risk CKD in KDIGO classification |
|  |  | The criterion is met | No clinical CVD |
| Stage 4:  Clinical CVD in CKM | Clinical CVD among individuals with excess/dysfunctional adiposity, other metabolic risk factors, or CKD | The criterion is met | Clinical CVD |
|  |  | Any of the nine criteria is met | Overweight/obesity |
|  |  |  | Abdominal obesity |
|  |  |  | Prediabetes |

**Supplementary Table 3. Detailed algorithm of the simplified 10-year CVD risk models.**

| **Total CVD (Base 10 year)** | |
| --- | --- |
| **Women** | **log-Odds** = -3.307728 + 0.7939329 × (age – 55) /10 + 0.0305239 × ((TC – HDL) × 0.02586 – 3.5) – 0.1606857 × (HDL × 0.02586 – 1.3) /0.3 – 0.2394003 × (min(SBP, 110) – 110) /20 + 0.360078 × (max(SBP, 110) – 130) /20 + 0.8667604 × (if diabetes) + 0.5360739 × (if current smoker) + 0.6045917 × (min(eGFR, 60) – 60) / -15 + 0.0433769 × (max(eGFR, 60) – 90) / -15 + 0.3151672 × (if using anti-hypertensive medication) – 0.1477655 × (if using statin) – 0.0663612 × (if using anti-hypertensive medication) × (max(SBP, 110) – 130) /20 + 0.1197879 × (if using statin) × ((TC – HDL) × 0.02586 – 3.5) – 0.0819715 × (age – 55) /10 × ((TC – HDL) × 0.02586 – 3.5) + 0.0306769 × (age – 55) /10 × (HDL × 0.02586 × 0.02586 – 1.3) /0.3 – 0.0946348 × (age – 55) /10 × (max(SBP, 110) – 130) /20 – 0.27057 × (age – 55) /10 × (if diabetes) – 0.078715 × (age – 55) /10 × (if current smoker) – 0.1637806 × (age – 55) /10 × (min(eGFR, 60) – 60) / -15  **Risk** = exp(log-Odds) / (1 + exp(log-Odds)) |
| **Men** | **log-Odds** = -3.031168 + 0.7688528 × (age – 55) /10 + 0.0736174 × ((TC – HDL) × 0.02586 – 3.5) – 0.0954431 × (HDL × 0.02586 – 1.3) /0.3 – 0.4347345 × (min(SBP, 110) – 110) /20 + 0.3362658 × (max(SBP, 110) – 130) /20 + 0.7692857 × (if diabetes) + 0.4386871 × (if current smoker) + 0.5378979 × (min(eGFR, 60) – 60) / -15 + 0.0164827 × (max(eGFR, 60) – 90) / -15 + 0.288879 × (if using anti-hypertensive medication) – 0.1337349 × (if using statin) – 0.0475924 × (if using anti-hypertensive medication) × (max(SBP, 110) – 130) /20 + 0.150273 × (if using statin) × ((TC – HDL) × 0.02586 – 3.5) – 0.0517874 × (age – 55) /10 × ((TC – HDL) × 0.02586 – 3.5) + 0.0191169 × (age – 55) /10 × (HDL × 0.02586 – 1.3) /0.3 – 0.1049477 × (age – 55) /10 × (max(SBP, 110) – 130) /20 – 0.2251948 × (age – 55) /10 × (if diabetes) – 0.0895067 × (age – 55) /10 × (if current smoker) – 0.1543702 × (age – 55) /10 × (min(eGFR, 60) – 60) / -15  **Risk** = exp(log-Odds) / (1 + exp(log-Odds)) |

**Supplementary Table 4. Assessment of multicollinearity of features using variance inflation factors (VIF).**

| **Features** | **VIF** |
| --- | --- |
| **Gender** | 1.2 |
| **Age** | 1.4 |
| **Race** | 1.1 |
| **Education level** | 1.3 |
| **Marital status** | 1.1 |
| **PIR** | 1.3 |
| **Smoking status** | 1.1 |
| **Alcohol** | 1.2 |
| **β-carotene** | 1.2 |
| **α-tocopherol** | 1.4 |
| **Lycopene** | 1.2 |
| **Folate** | 1.2 |
| **Vitamin C** | 1.3 |
| **25 (OH) D** | 1.2 |

**Supplementary Table 5.** **Hyperparameter for machine learning models.**

| **Models** | **Hyperparameter** |
| --- | --- |
| **RF** | max_depth: 6; min_samples_leaf: 1; n_estimators: 200. |
| **XGB** | max_depth: 6; n_estimators: 100; learning_rate: 0.1. |
| **CB** | max_depth: 6; learning_rate: 0.1; iterations: 100. |
| **LGBM** | max_depth: 6; learning_rate: 0.1; num_leaves: 51. |
| **DT** | max_depth: 3; min_samples_leaf: 1; min_samples_split: 2. |
| **KNN** | n_neighbors: 7; weights: distance. |
| **NB** |  |

**Supplementary Table 6.** **The calculation formulas for the machine learning model metrics.**

| **Metrics** | **Formula** |
| --- | --- |
| **Accuracy** | $\frac{TP+TN}{TP+TN+FP+FN}$ |
| **Precision** | $\frac{TP}{TP+FP}$ |
| **Sensitive/Recall** | $\frac{TP}{TP+FN}$ |
| **Specificity** | $\frac{TN}{FP+TN}$ |
| **F1 score** | $\frac{2*precision*recall}{precision+recall}$ |
| **FPR** | $\frac{FP}{FP+TN}$ |
| **FNR** | $\frac{FN}{TP+FN}$ |
| **PPV** | $\frac{TP}{FP+TP}$ |
| **NPV** | $\frac{TN}{FN+TN}$ |

**Supplementary Table 7. Confusion matrix for classification of advanced CKM.**

|  |  | **Predicted Class** | |
| --- | --- | --- | --- |
|  |  | Non-advanced CKM syndrome | Advanced CKM syndrome |
| **Actual Class** | Non-advanced CKM syndrome | True Negative (TN) | False Positive (FP) |
|  | Advanced CKM syndrome | False Negative (FN) | True Positive (TP) |

**Supplementary Table 8. PIPs of each serum micronutrients for the prevalence of advanced CKM syndrome in BKMR model.**

| **Features** | **Advanced CKM syndrome** |
| --- | --- |
|  | **PIPs** |
| **β-carotene** | 0.371 |
| **α-tocopherol** | 0.038 |
| **Lycopene** | 0.289 |
| **Folate** | 1.000 |
| **Vitamin C** | 0.013 |
| **25 (OH) D** | 0.390 |

**Supplementary Table 9. Mean SHAP values.**

| **Features** | **Mean SHAP values** |
| --- | --- |
| **Age** | 3.032098 |
| **Gender** | 0.653445 |
| **Alcohol** | 0.323439 |
| **Education level** | 0.234907 |
| **25 (OH) D** | 0.208280 |
| **PIR** | 0.168835 |
| **Vitamin C** | 0.168020 |
| **β-carotene** | 0.162313 |
| **α-tocopherol** | 0.148763 |
| **Lycopene** | 0.156762 |
| **Folate** | 0.139606 |
| **Race** | 0.128725 |
| **Marital status** | 0.153144 |
| **Smoking status** | 0.096627 |
